# Dual functionality of pathogenesis-related proteins: defensive role in plants versus immunosuppressive role in pathogens

**DOI:** 10.3389/fpls.2024.1368467

**Published:** 2024-08-02

**Authors:** Zhu Han, Roger Schneiter

**Affiliations:** Department of Biology, University of Fribourg, Fribourg, Switzerland

**Keywords:** plant immunity, fungal pathogens, secretion, apoplast, virulence, immune signaling, sperm coating proteins (SCPs), venom allergen-like proteins (VALs/VAPs)

## Abstract

Plants respond to pathogen exposure by activating the expression of a group of defense-related proteins known as Pathogenesis-Related (PR) proteins, initially discovered in the 1970s. These PR proteins are categorized into 17 distinct families, denoted as PR1-PR17. Predominantly secreted, most of these proteins execute their defensive roles within the apoplastic space. Several PR proteins possess well-defined enzymatic functions, such as β-glucanase (PR2), chitinases (PR3, 4, 8, 11), proteinase (PR7), or RNase (PR10). Enhanced resistance against pathogens is observed upon PR protein overexpression, while their downregulation renders plants more susceptible to pathogen infections. Many of these proteins exhibit antimicrobial activity *in vitro*, and due to their compact size, some are classified as antimicrobial peptides. Recent research has unveiled that phytopathogens, including nematodes, fungi, and phytophthora, employ analogous proteins to bolster their virulence and suppress plant immunity. This raises a fundamental question: how can these conserved proteins act as antimicrobial agents when produced by the host plant but simultaneously suppress plant immunity when generated by the pathogen? In this hypothesis, we investigate PR proteins produced by pathogens, which we term “PR-like proteins,” and explore potential mechanisms by which this class of virulence factors operate. Preliminary data suggests that these proteins may form complexes with the host’s own PR proteins, thereby interfering with their defense-related functions. This analysis sheds light on the intriguing interplay between plant and pathogen-derived PR-like proteins, providing fresh insights into the intricate mechanisms governing plant-pathogen interactions.

## Introduction

Plants are constantly challenged by various organisms, including fungi, oomycetes, bacteria, and viruses, which can compromise the plant’s fitness and survival (Teixeira et al., 2019). Plant pathogens affect forest plantations and most staple crops, decreasing productivity worldwide and severely compromising food security (Fones et al., 2020). The situation is expected to get worse, given the current rate of growth of the human population, the effect of climate change, the prevalence of monocultures, and the rise in pathogen resistance (Singh et al., 2023).

To combat the incursion of pathogens, plants have developed an intricate defense strategy comprising both inherent and inducible mechanisms (Jones and Dangl, 2006; Han, 2019). Constitutive defenses, operating as the foremost line of protection, encompass features like cutin, waxes, robust lignin deposition on cell walls, and the synthesis of antimicrobial small molecules, such as phytoanticipins (Li et al., 2020). The inducible defense mechanisms can be broadly categorized into two main types: Pathogen-associated molecular pattern (PAMP)-triggered immunity (PTI) and effector-triggered immunity (ETI). Furthermore, plants can develop systemic acquired resistance (SAR), a sophisticated response that fortifies defense throughout the plant following localized pathogen attack (Zhou and Zhang, 2020; Tanaka and Heil, 2021; Ngou et al., 2022).

## Pathogenesis-related proteins

Amidst the spectrum of plant defense mechanisms, PR proteins stand as a prominent line of primary defense. These proteins are categorized into various families, denoted as PR1 to PR17 and beyond, based on their unique structural and functional characteristics (van Loon and van Kammen, 1970). Typically, PR proteins are induced in response to pathogen invasion and complement the action of small organic defense compounds that primarily serve to fend off herbivores but also exhibit antimicrobial activities (Westrick et al., 2021).

The discovery of PR proteins traces back to pioneering studies in the 1970s, where their robust induction in response to tobacco mosaic virus infection was first observed (Gianinazzi et al., 1970; van Loon and van Kammen, 1970). Subsequent research extended this finding to various plant species facing diverse pathogens, including oomycetes, fungi, bacteria, viruses, viroids, nematodes, and insect pests (van Loon et al., 1987; Stintzi et al., 1993; Van Loon and Van Strien, 1999; Edreva, 2005; van Loon et al., 2006; Jain and Khurana, 2018; Zribi et al., 2021). The transcripts encoding PR proteins show rapid accumulation following PTI and ETI, with their expression often regulated by the signaling molecule salicylic acid (SA). Notably, PR1 proteins are distinguished as crucial molecular markers for heightened plant defense due to the induction of SAR (Vlot et al., 2009).

PR proteins exhibit distinct biochemical properties, such as low molecular weight (ranging from 6 to 43 kDa), extractability and stability at low pH (below 3, a condition under which most other proteins denature), thermostability, and resistance to proteases (Van Loon and Van Strien, 1999). They are found throughout various plant organs, with leaves being particularly rich in these proteins, where they can constitute up to 5-10% of total leaf proteins. The PR1 family, for example, can comprise 1-2% of total leaf proteins (Van Loon and Van Strien, 1999). In plants, multiple genes usually represent each PR protein family, enabling the synthesis of diverse protein isoforms. For example, *Arabidopsis thaliana* has 22 genes encoding PR1 homologs, and rice contains 39 PR1-type genes (Mitsuhara et al., 2008). Some of these PR1 genes are constitutively expressed in roots or floral tissues, implying roles in plant development. This wide distribution of defense-related proteins across monocots and dicots underscores their multifaceted functions beyond defense (van Loon et al., 2006).

PR proteins can be categorized based on their isoelectric points, with acidic variants primarily induced upon immune activation and secreted to the apoplast. In contrast, those with a basic isoelectric point are often involved in developmental processes, showing limited induction upon pathogen infection, and typically localizing intracellularly, particularly in vacuoles (Farvardin et al., 2020; Zribi et al., 2021). Certain PR proteins also respond to various abiotic stressors like wounding, dehydration, salt, or cold stress, while others possess anti-freeze activity, reflecting their roles under adverse environmental conditions (Griffith and Yaish, 2004; Islam et al., 2023). Importantly, several PR proteins present in pollen, fruits, and vegetables can trigger allergic reactions in humans, making them significant contributors to plant allergens (Arora et al., 2020).

Over the past five decades, extensive research has been dedicated to characterizing individual PR proteins, elucidating their basic enzymatic activities, and establishing their direct role in defense against microbial pathogens (van Loon et al., 2006; Ferreira et al., 2007; Ali et al., 2018; Dos Santos and Franco, 2023) (see **Table 1**). For instance, PR1 proteins exhibit lipid-binding activity and inhibit the growth of sterol auxotrophic oomycetes (Gamir et al., 2017; Han et al., 2023). PR1 proteins also harbor a C-terminal peptide known as CAP-derived peptide 1 (CAPE1), which, when cleaved from the full-length PR1 protein, stimulates plant immune defense (Chen et al., 2014; Breen et al., 2017; Chen et al., 2023). PR2 proteins share sequence homology with β-1,3-glucanases and can hydrolyze β-1,3-glucans, which are present in the cell walls of microbes, generating oligomers that serve as elicitors. PR3, PR8, and PR11 exhibit chitinase activity, often synergizing with PR2, and PR4 binds chitin, a key component of fungal cell walls (Levy et al., 2007; Balasubramanian et al., 2012; Perrot et al., 2022). PR5 encompasses thaumatin-like proteins (TLPs) that exert antimicrobial activity by rapidly permeabilizing microbial plasma membranes (Zhang et al., 2018b; de Jesús-Pires et al., 2020; Sharma et al., 2021). PR6 encodes a protease inhibitor and shows synergy with thionins (PR13) (Ryan, 1989; Terras et al., 1993; Sels et al., 2008; Grosse-Holz and van der Hoorn, 2016; Rawlings et al., 2018). PR7 encodes a subtilisin-like endoprotease, believed to attack and degrade microbial cell wall proteins. However, these proteolytic enzymes are also important for peptide signaling, for example, by releasing serine rich endogenous peptides (SCOOPs) in *Brassicacea*, which are then perceived by the leucine-rich repeat receptor kinase male discovery 1-interacting receptor-like kinase 2 (MIK2) to elicit immunity (Yang et al., 2023). PR9 exhibits heme-dependent peroxidase activity, crucial for lignification, wound healing, and oxidative degradation of phenolic compounds (Passardi et al., 2004; Almagro et al., 2009; Liu et al., 2018; Cesarino, 2019). PR10 proteins are members of the major latex-like family and have been reported to possess ribonuclease activity, but this might be attributed to copurifying RNase contaminations (Fernandes et al., 2013; Aglas et al., 2020; Longsaward et al., 2023). PR10 has a hydrophobic cavity capable of binding various lipids, including steroids and fatty acids (Radauer et al., 2008). Intriguingly, PR10 members are localized in the cytoplasm, but secreted into the apoplastic space when complexed with and activated by leucine-rich repeat protein 1 (LRR1) (Choi et al., 2012).

**Table 1:** Summary of properties of PR protein families.

Family	Pfam	Activity	Function/Properties	References*
PR1	PF00188	Immune signaling, Lipid-binding	• Antimicrobial • Abundant induced protein in the apoplast	(Chen et al., 2014; Breen et al., 2017; Gamir et al., 2017; Han et al., 2023)
PR2	PF00332	β-1,3-glucanase	• Antimicrobial • Cell wall degradation	(Levy et al., 2007; Balasubramanian et al., 2012; Perrot et al., 2022)
PR3 PR4 PR8 PR11	PF00182 PF00967 PF00704 PF00704	Chitinase (GH19) Chitin binding Chitinase (GH18) Chitinase (GH18)	• Antimicrobial • Cell wall degradation • Synergistic with PR2	(Oyeleye and Normi, 2018; Fukamizo and Shinya, 2019; Poria et al., 2021)
PR5	PF00314	Thaumatin/ Osmotin/ Zeamatin-like	• Antifungal • Glucan binding • Plasma membrane permeability • Sweet tasting • Anti-freeze activity	(Zhang et al., 2018; de Jesús-Pires et al., 2020; Sharma et al., 2021)
PR6	PF00280	Protease inhibitor MEROPS family	• Nematocidal • Insecticidal • Synergistic with PR13	(Terras et al., 1993; Ryan, 1989; Sels et al., 2008; Grosse-Holz and van der Hoorn, 2016; Rawlings et al., 2018)
PR7	PF00082	Subtilisin-like endoprotease	• Antifungal • Dissociation of microbial cell wall •Phytocytokine signaling	(Figueiredo et al., 2018; Schaller et al., 2018; Yang et al., 2023)
PR9	PF00141	Heme-containing peroxidase	• Lignin-forming peroxidase	(Passardi et al., 2004; Almagro et al., 2009; Liu et al., 2018; Cesarino, 2019)
PR10	PF00407	Ribonuclease-like, large hydrophobic cavity	• Antimicrobial • Cytoplasmic protein • Related to Bet v 1, a major birch pollen allergen	(Radauer et al., 2008; Choi et al., 2012; Fernandes et al., 2013; Aglas et al., 2020)
PR12	PF00304	Plant defensin	• Antimicrobial • Induction of ion efflux • Interaction with fungal sphingolipids	(Terras et al., 1995; Thevissen et al., 2000; Sels et al., 2008; Tam et al., 2015; Parisi et al., 2019)
PR13	PF00321	Thionin	• Antimicrobial • Membrane permeating • Synergistic with PR14	(Stec, 2006; Sels et al., 2008; Tam et al., 2015; Höng et al., 2021)
PR14	PF00234	Non-specific lipid-transfer protein Protease inhibitor Seed storage	• Antimicrobial	(Sels et al., 2008; Liu et al., 2015; McLaughlin et al., 2021; Gao et al., 2022; Melnikova et al., 2022)
PR15 PR16	PF00190 PF00190	Oxalate oxidase Oxalate oxidase-like	• Antimicrobial • ROS generation • Germin • Cupin family	(Mittler, 2002; Dunwell et al., 2004; Farvardin et al., 2020; Joshi et al., 2021)
PR17	PF04450	Putative aminopeptidase	• Poorly characterized	(Okushima et al., 2000; Christensen et al., 2002; Joshi et al., 2021)

*We predominantly reference review articles in this table, aiming to provide a comprehensive overview of the individual members within the PR class of proteins. This approach is taken due to the extensive nature of the original literature encompassing these 17 distinct protein families, spanning over 50 years, and involving numerous plant species as well as specific types of pathogen interactions. Notably, the PR18 and PR19 proteins, although recently incorporated, are omitted from this compilation. This omission arises from their limited characterization thus far, with their enzymatic activity and mode of action yet to be elucidated (Ali et al., 2018).

PR12 comprises plant defensins, small proteins with antimicrobial activity but an uncharacterized mode of action (Terras et al., 1995; Thevissen et al., 2000; Sels et al., 2008; Tam et al., 2015; Parisi et al., 2019). PR6, PR12, PR13, and PR14, due to their low molecular weight and antimicrobial activity, are classified as antimicrobial peptides (Sels et al., 2008). PR13 belongs to the class of thionins, small, basic, and cysteine-rich peptides that, like PR12 peptides, cause the permeabilization of microbial cell membranes. PR13 exhibits synergistic antimicrobial activity with PR14 (Stec, 2006; Sels et al., 2008; Tam et al., 2015; Höng et al., 2021). PR14 proteins can transfer phospholipids between membranes *in vitro* and, due to their low substrate specificity, are known as non-specific lipid transfer proteins (ns-LTPs) (Sels et al., 2008; Liu et al., 2015; Gao et al., 2022; Melnikova et al., 2022). PR15 and PR16, oxalate oxidase and oxalate oxidase-like proteins, contribute to the generation of apoplastic reactive oxygen species (ROS), initiating signal transduction cascades and activating plant defense mechanisms (Mittler, 2002; Dunwell et al., 2004; Farvardin et al., 2020; Joshi et al., 2021). Lastly, PR17, the least understood class, is postulated to possess aminopeptidase activity (Okushima et al., 2000; Christensen et al., 2002; Joshi et al., 2021).

Numerous PR proteins display antimicrobial activity *in vitro*, and their overexpression in plants enhances resistance to various pathogens across diverse plant species (Alexander et al., 1993; Niderman et al., 1995; Epple et al., 1997; Anand et al., 2004; van Loon et al., 2006; Ferreira et al., 2007; Sels et al., 2008; Dos Santos and Franco, 2023). Conversely, silencing the expression of PR1 or PR5 renders plants more susceptible to pathogens (Riviere et al., 2008; Zhang et al., 2018b). Despite their antimicrobial activity, the precise functions of many PR proteins in defense responses remain incompletely understood, extending beyond direct pathogen inhibition to encompass roles in cell wall reinforcement, scavenging of ROS, and modulation of defense signaling pathways (van Loon et al., 2006; Islam et al., 2023). Given the protective effects conferred by the induction and accumulation of PR proteins, their overexpression, and heterologous expression are currently explored as strategies to establish stress-tolerant plants (Ali et al., 2018; Boccardo et al., 2019; Islam et al., 2023).

## Pathogenesis-related-like proteins produced by pathogens

While PR proteins are typically produced by plants in response to pathogen infection as part of their defense mechanism, recent findings have unveiled a fascinating twist: pathogens themselves synthesize pathogenesis-related-like proteins, which we will refer to as PR-like proteins, that play crucial roles in promoting pathogen virulence (Han et al., 2023). Unlike the induction and secretion of antimicrobial proteins by the host plant upon pathogen attack, which are well studied, the precise function and contribution of PR-like proteins to pathogen virulence remain enigmatic.

Among PR-like proteins, the PR1-like family is perhaps the most extensively characterized. PR1 proteins belong to a large protein superfamily, also known as CAP proteins (CRISP/Ag5/PR1) or SCPs (sperm coating proteins) and are related to VALs/VAPs (venom allergen-like proteins made by nematodes) (Gibbs et al., 2008; Cantacessi and Gasser, 2012; Wilbers et al., 2018; Han et al., 2023). Recent research has unveiled PR1-like proteins from various pathogenic nematodes and fungi as novel virulence factors. For example, PR1-like proteins from hemibiotrophic *Fusarium* species, including Fpr1 from *Fusarium oxysporum*, FgPR1L-4 from *Fusarium graminearum*, as well as FvSCP1 from *Fusarium verticillioides*, have all been shown to enhance fungal virulence in their respective host plants (Prados-Rosales et al., 2012; Lu and Edwards, 2018; Zhang et al., 2018a). More recently, a family of three highly related PR1-like proteins was identified in the necrotrophic fungal pathogens *Cytospora chrysosperma* and *Valsa mali*, causal agents of canker disease in poplar and apple, respectively. Deletion of CcCAP1 in *C. chrysosperma* reduced fungal virulence and increased sensitivity to ROS, highlighting its importance (Han et al., 2021). Additionally, two of the three *V. mali* PR1-like proteins, VmPR1a and VmPR1c, were found to be essential for pathogen virulence (Wang et al., 2021). Recent host-induced gene silencing experiments further demonstrated that three out of six PR1-like proteins from the wheat stripe rust fungus *Puccinia striiformis* f. sp. *tritici* are necessary for fungal virulence (Zhao et al., 2023).

Furthermore, in susceptible tomato plants, GrVAP1 secreted by the potato cyst nematode (*Globodera rostochiensis*) is required for successful infection (Lozano-Torres et al., 2014). However, in other cultivars, GrVAP1 interacts with the tomato papain-like cysteine protease Rcr3, activating the membrane-localized immune receptor Cf-2, thereby inducing the host’s immune response (Lozano-Torres et al., 2012). Similarly, a PR1-like protein from *Phytophthora sojae* has been found to trigger an immune response in *Nicotiana benthamiana*, dependent on its recognition by the leucine-rich repeat receptor-like protein (LRR-RLP) RCAP1. This recognition involves the shared immune coreceptors BAK1 and SOBIR1 and leads to increased plant resistance against Phytophthora (Gust and Felix, 2014; Liebrand et al., 2014; Jiang et al., 2023). PsCAP1, the Phytophthora PR1-like protein, contains an N-terminal PAN domain and exhibits immune-stimulatory activities such as triggering ROS bursts, activating mitogen-activated protein kinase (MAPK), and inducing cell death. Importantly, these activities are mediated by the PAN domain, which is distinct from the CAP domain found in canonical PR1 proteins. The PAN domain has been proposed to facilitate protein-protein and protein-carbohydrate interactions, but its precise role in plant-microbe interactions remains a subject of study (Jiang et al., 2023). This PAN domain containing PsCAP1 protein is conserved among phytopathogenic oomycetes but absent in the genomes of plants, diatoms, bacteria, or fungi (Jiang et al., 2023).

Interestingly, heterologous expression of PR1-like proteins from pathogens, such as GrVAP1 from *G. rostochiensis* or CcCAP1 from *C. chrysosperma*, in host plants suppresses the plant’s PTI response. Expression of CcCAP1 in tobacco inhibits pathogen-induced induction of PR1 and PR4 and the expression of GrVAP1 selectively suppresses the activation of the programmed cell death by surface-localized immune receptors (Lozano-Torres et al., 2014; Han et al., 2021). These observations suggest that these proteins possess potent immune modulatory activity, rendering plants hypersensitive to various unrelated pathogens (Lozano-Torres et al., 2014; Han et al., 2021).

The corn smut *Ustilago maydis* UmPR1-like protein has recently been shown to sense plant-derived phenolic compounds to eliciting hyphal-like growth to guide fungal invasion in plants. In addition, secretion of UmPR1-like promotes fungal virulence by hijacking a plant cysteine protease to release a UmCAPE-like signaling peptide from UmPR1-like and suppress plant immunity (Lin et al., 2023).

These PR1-like proteins from pathogens appear to function in ways similar to plant hormones produced by pathogenic and symbiotic fungi. They may have dual roles: (i) perturbing plant processes, either positively or negatively, to promote invasion and nutrient uptake by the pathogens; and (ii) serving as signals for the fungi to engage in appropriate developmental and physiological processes adapted to their environment (Chanclud and Morel, 2016).

Building upon these insights into PR1-like proteins, we explored the genomes of various plant pathogens for the presence of other PR-like genes. Remarkably, we found that not only PR1-like genes are prevalent in phytopathogen genomes but that many other PR-like protein family members are also present. Except for PR4 (chitinase), PR6 (protease inhibitor), PR10 (ribonuclease-like), PR12 (plant defensin), PR13 (thionin), and PR14 (non-specific lipid transfer protein), multiple copies of genes encoding PR-like proteins are frequently identified in the genomes of model phytopathogens, particularly in those of fungi and oomycetes (Dean et al., 2012; Kamoun et al., 2015). Notable examples include the rice blast fungus *Magnaporthe oryzae* (Tan et al., 2023), the gray mold fungus *Botrytis cinerea* (Bi et al., 2023), the rust fungus *Puccinia* spp (Avasthi et al., 2023), the soil-borne ascomycete *Fusarium oxysporum* (Srinivas et al., 2019), the causative agent of wilt disease *Verticillium dahliae* (Klosterman et al., 2009), the corn smut fungus *Ustilago maydis* (Yu et al., 2023), as well as the oomycetes *Phytophthora infestans* (Whisson et al., 2016), which cause late blight disease on potato and tomato, the downy mildew causing *Hyaloperonospora arabidopsidis* (Coates and Beynon, 2010), and the sudden oak death disease causing *Phytophthora ramorum* (Grünwald et al., 2008) (see **Table 2**; **Supplementary Materials Table S1**). These intriguing observations suggests that the phenomena described for PR1-like proteins likely extend to other PR-like protein families as well. Consequently, some of the key questions that arise are: What functions do these PR-like proteins serve in phytopathogens? Do their deletions impact pathogen virulence? Can their heterologous expression in plants render them more susceptible to a broader spectrum of pathogens? What are the mechanisms of action employed by pathogen-produced PR-like proteins?

**Table 2:** Genes Encoding PR-Like Proteins in Filamentous Phytopathogens.

Family	Pfam	Activity	*Magnaporthe oryzae*	*Botrytis cinerea*	*Puccinia* spp.	*Fusarium oxysporum* f.sp.	*Verticillium dahliae*	*Ustilago maydis*	*Phytoph-thora infestans*	*Hyalope-ronospora arabidopsidis*	*Phytophthora ramorum*	*Key References*
PR1	PF00188	Immune signaling Lipid-binding	6	4	6	7	4	2	29	13	21	(Han et al., 2023; Lin et al., 2023; Jiang et al., 2023; Zhao et al., 2023; Han et al., 2021; Lozano-Torres et al., 2014; Teixeira et al., 2012; Prados-Rosales et al., 2012)
PR2	PF00332	β-1,3-glucanase	3	4	2	4	6	2	7	3	7	(Sarthy et al., 1997; Chen et al., 2017)
PR3	PF00182	Chitinase (GH19)	/	/	/	/	/	/	3	1	1	(Bělonožníková et al., 2022; Guo et al., 2023)
PR4	PF00967	Chitin-binding	/	/	/	/	/	/	/	/	/	
PR5	PF00314	Thaumatin	1	2	4	1	1	1	2	/	/	(Meng et al., 2019; Kirino et al., 2020)
PR6	PF00280	Protease inhibitor	/	/	/	/	/	/	/	/	/	
PR7	PF00082	Subtilisin-like endoprotease	30	13	16	32	18	5	13	6	7	(Monod et al., 2002; Shi et al., 2014; Xu et al., 2020; Liu et al., 2020)
PR8/ PR11	PF00704	Chitinase (GH18))	16	7	15	28	18	3	2	2	2	(Han et al., 2019; Bradley et al., 2022; Bělonožníková et al., 2022; Guo et al., 2023)
PR9	PF00141	Heme-containing peroxidase	10	4	2	10	7	3	4	4	6	(Mir et al., 2015)
PR10	PF00407	Ribonuclease-like	/	/	/	/	/	/	/	/	/	
PR12	PF00304	Plant defensin	/	/	/	/	/	/	/	/	/	
PR13	PF00321	Thionin	/	/	/	/	/	/	/	/	/	
PR14	PF00234	Non-specific lipid-transfer protein	/	/	/	/	/	/	/	/	/	
PR15/ PR16	PF00190	Oxalate oxidase Oxalate oxidase-like	1	3	/	7	6	/	/	/	/	(El Hadrami et al., 2015; Liang et al., 2015; Fan et al., 2021; Yan et al., 2022)
PR17	PF04450	Putative aminopeptidase	1	1	/	1	1	/	/	/	/	

The table gives an overview of the number of PR-like proteins that are present in fungal (left-hand part) and oomycetes (right-hand part) phytopathogens. /, indicates that there is no gene present that matches the annotation of the respective plant PR protein family. PR-like families that are absent from the genomes of fungal or oomycetes are shaded in light green, i.e., PR3-, PR4-, PR6-, PR10-, PR12-, PR13-, PR14-, PR17-like. Genes were identified by screening sequences of individual plant PR family members against the genome sequences of *Magnaporthe oryzae* (*Pyricularia oryzae* 70-15, v3.0), *B. cinerea* (B05.10), *Puccinia* spp. (*Puccinia striiformis* f.sp. *tritici* PST-78, v1.0), *Fusarium oxysporum* **(** *Fusarium oxysporum* f.sp. *lycopersici* 4287, v2), *Verticillium dahliae* (VdLs.17), *Ustilago maydis* (521, v2.0), *Phytophthora infestans* (T30-4), *Hyaloperonospora arabidopsidis* (Emoy2, v2.0), and *Phytophthora ramorum* (v1.1) in the PhytoPath database (https://phytopathdb.org/) (Pedro et al., 2016). Gene identifiers for all these PR-like family members are provided in **Supplementary Materials, Table S1**. Gray shading is used to differentiate between rows.

**Figure 1:**
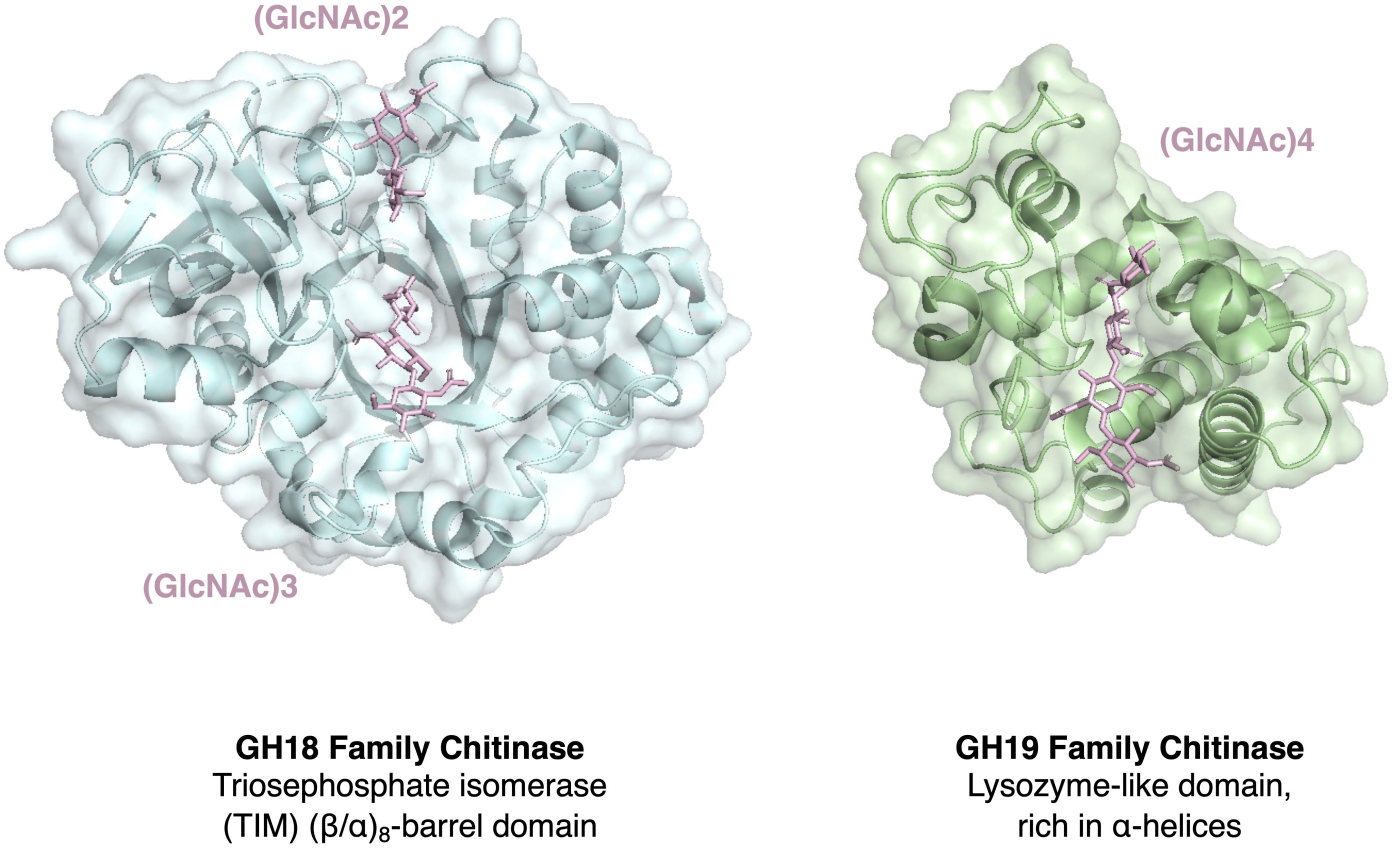
Comparison of the structure of chitinases belonging to two major families. Structural comparison of the ubiquitous chitinases belonging to the glycoside hydrolase family 18 (GH18) and the more plant-specific GH19 family. PR8 and PR11 are members of the bacterial/fungal GH18 family whereas PR3 is a member of the GH19 family and has structural similarity to some lysozymes (Monzingo et al., 1996). Shown are the structures of the triosephosphate isomerase (TIM) (β/α)_8_-barrel domain-containing GH18 family chitinase from *Cycas revoluta* in complex with the chitin dimer (GlcNAc)2 and chitin trimer (GlcNAc)3 (PDB 4MNK), and the α-helix-rich lysozyme-like GH19 family chitinase from *Bryum coronatum* in complex with the chitin tetramer (GlcNAc)4 (PDB 4IJ4; Ohnuma et al., 2014).

The role of PR-like proteins in pathogen vegetative growth, virulence, or their function as effectors modulating the host’s immune response are not well characterized, except for the cell wall remodeling enzymes including β-1,3-glucanases, such as Bgl2 in *Candida albicans*, and chitinases, which have established roles in filamentous growth, conidial germination, or haustorium establishment (Sarthy et al., 1997; Chen et al., 2017; Han et al., 2019; Guo et al., 2023). However, these cell wall remodeling enzymes may function primarily as morphogenetic factors rather than classical effectors, even though chitin can induce strong PTI, and its immune modulation involves processes such as shielding through lectin binding or deacetylation to chitosan (Gong et al., 2020). Interestingly, PR8 and PR11 chitinases belong the glycosyl hydrolase family 18 (GH18), a bacterial type of endochitinase, which are widely distributed in almost all organisms including plant pathogens (Bradley et al., 2022) (**Table 2**). PR3, on the other hand, belongs to the glycosyl hydrolase family 19 (GH19), which are mostly found in plants, and possess a specific chitin binding domain, which is absent in the bacterial type of enzyme (Henrissat and Bairoch, 1993). Members of this family are thought to be produced as part of a defense response against fungal pathogens. The overall structures and catalytic domains of these two classes of chitinase differ greatly. The GH19 family chitinases have an α-helix-rich lysozyme-like domain characterized by a deep cleft, whereas GH18 chitinases are characterized by a catalytic region that consists of a triosephosphate isomerase (TIM) (β/α)_8_-barrel domain (Oyeleye and Normi, 2018; Fukamizo and Shinya, 2019; Poria et al., 2021) (**Figure 1**). Interestingly, oomycetes express the GH19 plant type of chitinase as well, whereas most of the fungal pathogens do not (Klinter et al., 2019). This is particularly intriguing given that the cell wall of oomycetes is primarily composed of cellulose and β-glucans rather than chitin (Mélida et al., 2013; Wanke et al., 2021). These GH19 family chitinases in oomycetes have likely been acquired by horizontal gene transfer and been proposed to be important for the degradation of complex carbohydrates present in fungal cell walls during mycoparasitism (Liang et al., 2020; Bělonožníková et al., 2022).

On the other hand, silencing of the PR5-like thaumatin-like protein from the pine wood nematode *Bursaphelenchus xylophilus* has been shown to reduce the pathogen’s reproduction and pathogenicity. When expressed in tobacco, it induces a robust cell death response (Meng et al., 2019; Kirino et al., 2020; Meng et al., 2022). Thaumatin-like proteins have been reported to bind β-1,3-glucans, exhibit endo-β-1,3-glucanase activity, inhibit α-amylase, or permeabilize cell membranes, yet their precise antimicrobial mechanisms remain ambiguous (Roberts and Selitrennikoff, 1990; Abada et al., 1996; Trudel et al., 1998; Grenier et al., 1999; Koiwa et al., 1999; Franco et al., 2002; Menu-Bouaouiche et al., 2003; de Jesús-Pires et al., 2020; Sharma et al., 2021). Thaumatin-like proteins are found in fungi, nematodes, and insects but are absent in vertebrates (Brandazza et al., 2004; Sakamoto et al., 2006; Belaish et al., 2008; Meng et al., 2019; de Jesús-Pires et al., 2020; Kirino et al., 2020).

## Discussion

Several studies have highlighted the interactions between plant and fungal PR1 and PR1-like proteins, shedding light on their potential roles in modulating the host’s immune response. Notably, some of these proteins form homodimers, exemplified by the wheat protein TaPR1-5, Fpr1 from the soilborne fungal pathogen *F. oxysporum*, and *S. cerevisiae* Pry1 (Prados-Rosales et al., 2012; Lu et al., 2013; Darwiche et al., 2016). Furthermore, it has been shown that the dimeric form of TaPR1-5 is a specific target of ToxA, a host-selective virulence factor secreted by the causal agent of wheat tan spot disease, *Pyrenophora tritici-repentis* and the leaf/glume blotch fungus *Stagonospora nodorum* (*Sn*) (Lu et al., 2014). The binding of SnToxA to TaPR1-5 appears to compromise the immune-protective function of PR1 in wheat, thereby promoting necrosis (Ciuffetti et al., 2010).

Intriguingly, a second effector protein, SnTox3, secreted by *S. nodorum*, interacts with a broader range of wheat PR1 isoforms than SnToxA. SnTox3 effectively inhibits the release of CAPE1, thus suppressing the plant’s immune defense mechanisms (Breen et al., 2016; Sung et al., 2021). These findings suggest a multifaceted strategy by phytopathogens to subvert the host’s immune response, utilizing distinct effectors to target different components of the plant’s defense system.

Beyond the interactions with pathogenic effectors, PR1 has been shown to form heteromeric complexes with other PR proteins, particularly PR5 and PR14. The thaumatin-like PR5 is secreted into the apoplastic space and rapidly accumulates in response to various stressors, both biotic and abiotic (Hakim et al., 2018; Zhang et al., 2018b). Notably, wheat PR5 (TaTLP1) directly interacts with TaPR1, and the antimicrobial activity of the resulting heteromeric complex surpasses that of either PR5 or PR1-4 alone. This synergy suggests that these proteins act cooperatively to enhance the plant’s defense against invading pathogens (Wang et al., 2020, 2022).

On the other hand, PR14 belongs to the ns-LTP family. These extracellular ns-LTPs are known to bind to and transfer lipids between membranes *in vitro*. *In vivo*, they may serve as lipid sensors or sequester lipids to modulate their potential signaling functions (Missaoui et al., 2022). Wheat PR14 (TaLTP3) associates with TaPR1 in the apoplast, and plants overexpressing both proteins activate multiple signaling cascades, including the SA, jasmonic acid, and auxin pathways, and they exhibit enhanced production of ROS during the defense response. This interaction, together with the fact that purified PR14 exhibits antimicrobial activity *in vitro*, highlights the role of PR14 in reinforcing plant immunity (McLaughlin et al., 2021; Zhao et al., 2021).

These reported protein interactions suggest that the association between PR1-like proteins and their various PR family members may function to modulate plant-pathogen interactions. Given that PR1-like proteins from certain pathogens, such as the nematode *G. rostochiensis* (GrVAP1) or the fungal pathogen *C. chrysosperma* (CcCAP1), have been shown to reduce host immunity when expressed in plants (Lozano-Torres et al., 2014; Han et al., 2021), these PR1-like proteins may interfere with the immune-stimulating actions of endogenous PR1 proteins, similarly to the effectors SnToxA or SnTox3. This interference may occur through the disruption of protein complexes between plant’s own PR1 and PR5 and/or PR14 or by impeding the CAPE1-mediated signaling of PR1 (Breen et al., 2017; Han et al., 2023).

**Figure 2:**
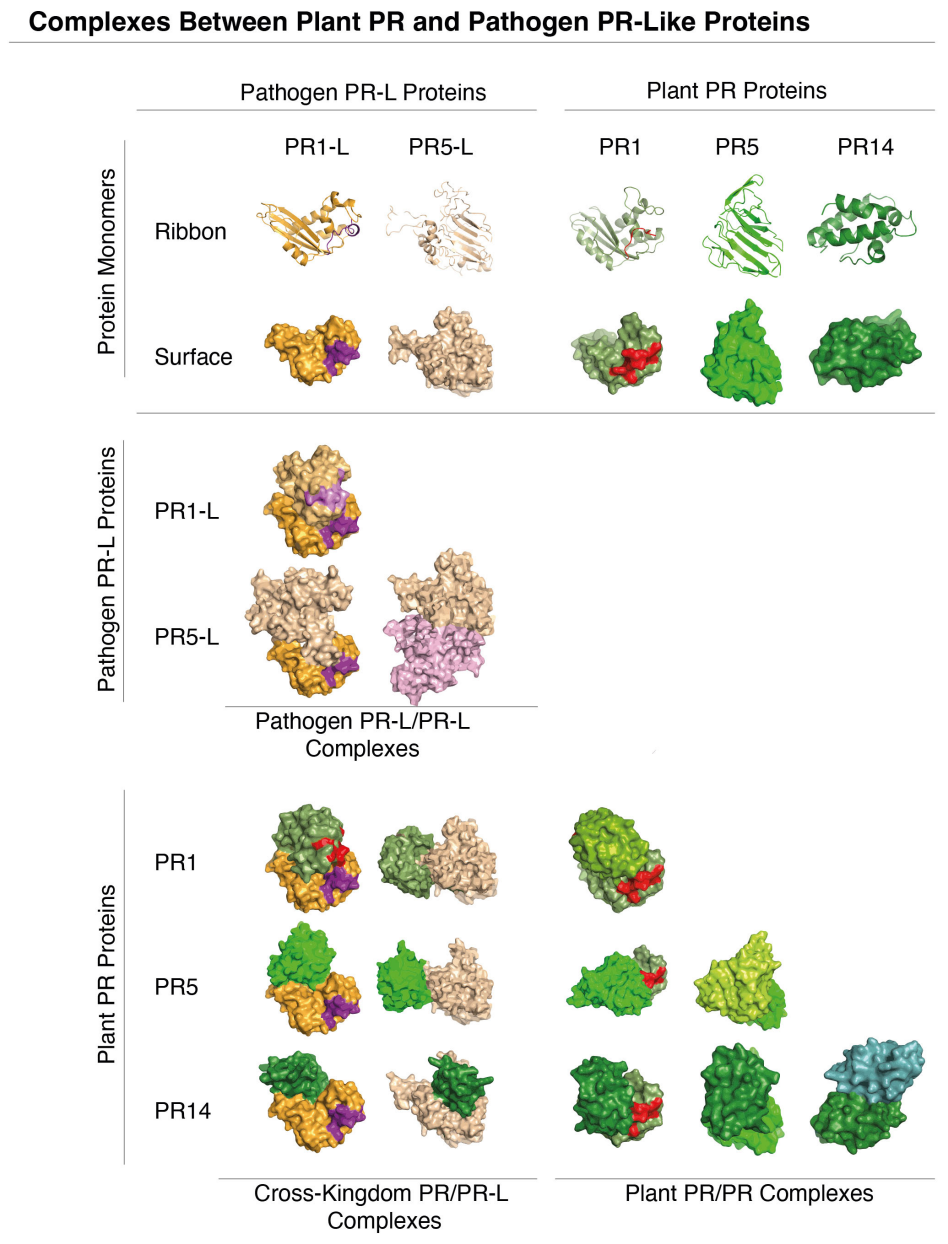
Molecular docking of phytopathogen PR1-like and PR5-like proteins with plant PR1, PR5 and PR14. Predicted molecular interactions between PR1-like and PR5-like proteins (orange) from phytopathogens and plant PR1, PR5, and PR14 (green) are visualized in the matrix. Notably, PR-like (PR-L) proteins from phytopathogens can interact both with themselves, and with host plant PR proteins. The docking simulations of PR1-like and PR5-like proteins from *Botrytis cinerea* with plant PR1, PR5, and PR14 (*Arabidopsis thaliana*) were performed using UCSF Chimera (Pettersen et al., 2004). The pathogen’s CAPE-like and the plant’s CAPE immune stimulatory peptides in the C-terminal end of PR1 and PR1-like are indicated in violet and red, respectively. Structures of monomeric proteins shown in the top two rows are represented both by ribbon diagrams and as space-filling models.

**Figure 3:**
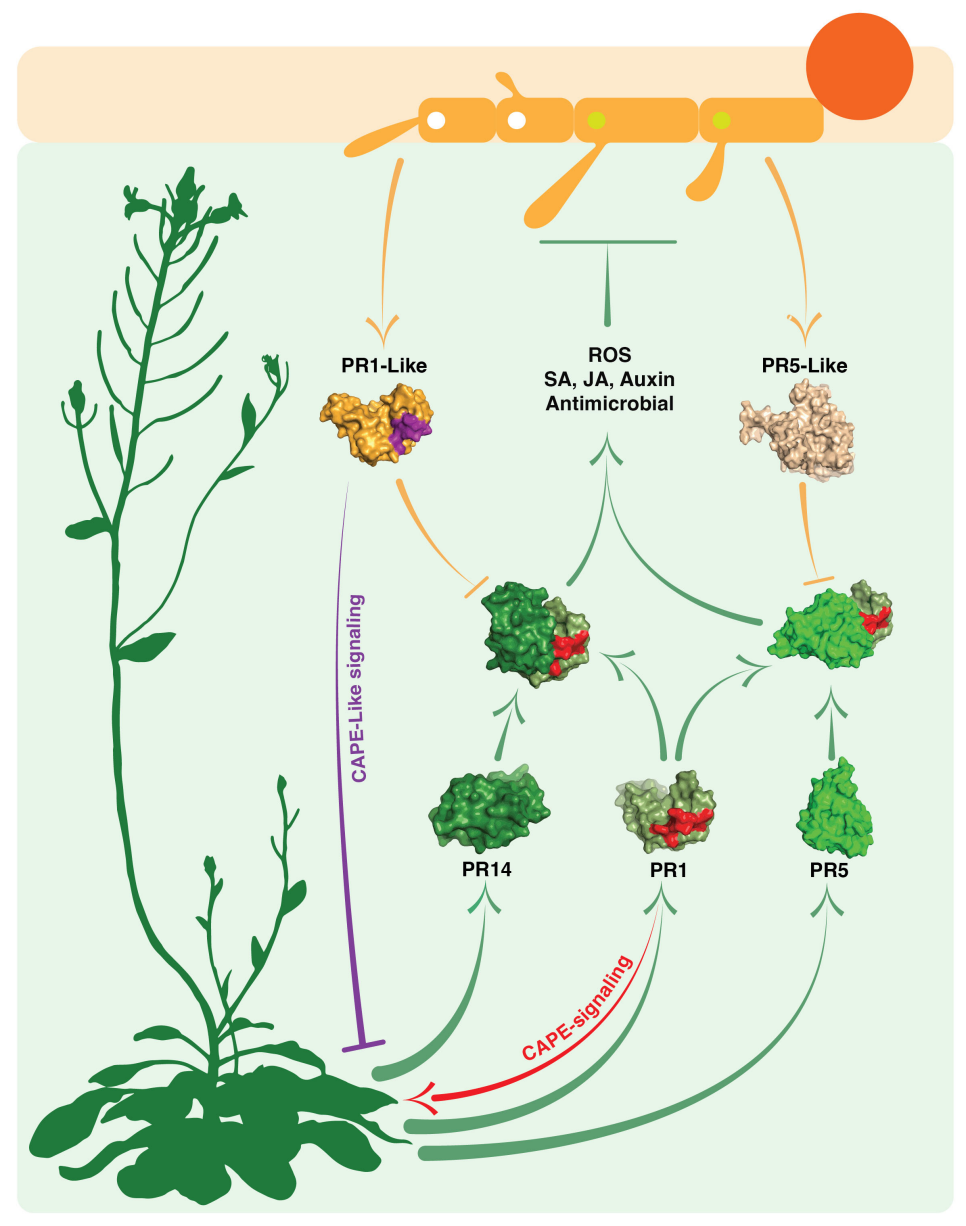
Interactions between plant defense-related PR proteins and phytopathogen PR-like proteins. This schematic diagram illustrates the complex interactions occurring in the apoplastic space of host plants (light green shaded space). It showcases the interplay between the host plant’s own PR1, PR5, and PR14 proteins (depicted by green arrows and green space-filling models) and the PR1-like and PR5-like proteins secreted by invading phytopathogens (depicted by orange arrows and orange space-filling models). The light green shading represents the host’s apoplastic space. The figure displays the structures of heteromeric complexes formed by plant PR1 with PR5 or PR14 proteins. The formation of these complexes contributes to immune stimulatory processes (involving SA, salicylic acid; JA, jasmonic acid; and auxin) as well as antimicrobial responses (including ROS production and direct antimicrobial activity). Importantly, the figure also suggests that these interactions can be disrupted by pathogen-derived PR-like proteins. Furthermore, it highlights CAPE immune stimulatory signaling by plant PR1 (indicated by the red arrow) and CAPE-like inhibitory signaling mediated by the pathogen’s PR1-like protein (indicated by the violet blunt arrow).

Consistent with the potential mode of action of PR-like proteins, *in silico* docking experiments suggest that PR1-like proteins from pathogens can indeed form protein complexes with plant endogenous PR5 and PR14, thus potentially undermining the host’s PR-based defense mechanisms (**Figure 2**). The prediction of protein complexes is emerging as a new powerful tool to identify potential microbial effectors. A recent bioinformatics screen using AlphaFold-Multimer, for example, has identified PR7, a subtilisin-like endoprotease PR7 (also known as P69 subtilase), as an effector hub targeted by different microbial kingdoms (Homma et al., 2023). This discovery lends further support to the notion that PR-like proteins may play pivotal roles in manipulating the plant’s immune response through the formation of cross-kingdom heteromeric protein complexes (**Figure 3**).

In conclusion, accumulating evidence suggests that phytopathogens have evolved strategies involving PR-like proteins to subdue their host’s immune response. These proteins appear to have coopted elements of the plant’s innate defense mechanisms, our hypothesis is that these proteins potentially interfere with the formation of protein complexes involving PR1, PR5, and PR14 within the apoplastic space and with key signaling pathways mediated by the CAPE peptide of PR1. While these findings offer valuable insights, it is important to emphasize that further experimental validation is necessary to establish the exact mechanisms underlying the interactions between PR and PR-like proteins and their impact on plant immunity.

## Data availability statement

The original contributions presented in the study are included in the article/**Supplementary Material**. Further inquiries can be directed to the corresponding author.

## Author contributions

ZH: Conceptualization, Data curation, Funding acquisition, Investigation, Validation, Visualization, Writing – original draft, Writing – review & editing. RS: Conceptualization, Funding acquisition, Project administration, Supervision, Validation, Visualization, Writing – original draft, Writing – review & editing.

## References

[B1] Abada L. R. D’Urzo M. P. Liua D. Narasimhan M. L. Reuveni M. Zhua J. K.. (1996). Antifungal activity of tobacco osmotin has specificity and involves plasma membrane permeabilization. Plant Sci. 118, 11–23. doi: 10.1016/0168-9452(96)04420-2

[B2] Aglas L. Soh W. T. Kraiem A. Wenger M. Brandstetter H. Ferreira F. (2020). Ligand binding of PR-10 proteins with a particular focus on the Bet v 1 allergen family. Curr. Allergy Asthma Rep. 20, 25. doi: 10.1007/s11882-020-00918-4 32430735 PMC7237532

[B3] Alexander D. Goodman R. M. Gut-Rella M. Glascock C. Weymann K. Friedrich L.. (1993). Increased tolerance to two oomycete pathogens in transgenic tobacco expressing pathogenesis-related protein 1a. Proc. Natl. Acad. Sci. U.S.A. 90, 7327–7331. doi: 10.1073/pnas.90.15.7327 8346252 PMC47130

[B4] Ali S. Ganai B. A. Kamili A. N. Bhat A. A. Mir Z. A. Bhat J. A.. (2018). Pathogenesis-related proteins and peptides as promising tools for engineering plants with multiple stress tolerance. Microbiol. Res. 212-213, 29–37. doi: 10.1016/j.micres.2018.04.008 29853166

[B5] Almagro L. Gómez Ros L. V. Belchi-Navarro S. Bru R. Ros Barceló A. Pedreño M. A. (2009). Class III peroxidases in plant defence reactions. J. Exp. Bot. 60, 377–390. doi: 10.1093/jxb/ern277 19073963

[B6] Anand A. Lei Z. Sumner L. W. Mysore K. S. Arakane Y. Bockus W. W.. (2004). Apoplastic extracts from a transgenic wheat line exhibiting lesion-mimic phenotype have multiple pathogenesis-related proteins that are antifungal. Mol. Plant Microbe Interact. 17, 1306–1317. doi: 10.1094/MPMI.2004.17.12.1306 15597736

[B7] Arora R. Kumar A. Singh I. K. Singh A. (2020). Pathogenesis related proteins: a defensin for plants but an allergen for humans. Int. J. Biol. Macromol 157, 659–672. doi: 10.1016/j.ijbiomac.2019.11.223 31790737

[B8] Avasthi S. Gautam A. K. Niranjan M. Verma R. K. Karunarathna S. C. Kumar A.. (2023). Insights into diversity, distribution, and systematics of rust genus puccinia. J. Fungi (Basel) 9, 639. doi: 10.3390/jof9060639 37367575 PMC10303085

[B9] Balasubramanian V. Vashisht D. Cletus J. Sakthivel N. (2012). Plant β-1,3-glucanases: their biological functions and transgenic expression against phytopathogenic fungi. Biotechnol. Lett. 34, 1983–1990. doi: 10.1007/s10529-012-1012-6 22850791

[B10] Belaish R. Sharon H. Levdansky E. Greenstein S. Shadkchan Y. Osherov N. (2008). The *Aspergillus nidulans* cetA and calA genes are involved in conidial germination and cell wall morphogenesis. Fungal Genet. Biol. 45, 232–242. doi: 10.1016/j.fgb.2007.07.005 17703972

[B11] Bělonožníková K. Hýsková V. Chmelík J. Kavan D. Čeřovská N. Ryšlavá H. (2022). Pythium oligandrum in plant protection and growth promotion: Secretion of hydrolytic enzymes, elicitors and tryptamine as auxin precursor. Microbiol. Res. 258, 126976. doi: 10.1016/j.micres.2022.126976 35158298

[B12] Bi K. Liang Y. Mengiste T. Sharon A. (2023). Killing softly: a roadmap of Botrytis cinerea pathogenicity. Trends Plant Sci. 28, 211–222. doi: 10.1016/j.tplants.2022.08.024 36184487

[B13] Boccardo N. A. Segretin M. E. Hernandez I. Mirkin F. G. Chacón O. Lopez Y.. (2019). Expression of pathogenesis-related proteins in transplastomic tobacco plants confers resistance to filamentous pathogens under field trials. Sci. Rep. 9, 2791. doi: 10.1038/s41598-019-39568-6 30808937 PMC6391382

[B14] Bradley E. L. Ökmen B. Doehlemann G. Henrissat B. Bradshaw R. E. Mesarich C. H. (2022). Secreted glycoside hydrolase proteins as effectors and invasion patterns of plant-associated fungi and oomycetes. Front. Plant Sci. 13. doi: 10.3389/fpls.2022.853106 PMC8960721 35360318

[B15] Brandazza A. Angeli S. Tegoni M. Cambillau C. Pelosi P. (2004). Plant stress proteins of the thaumatin-like family discovered in animals. FEBS Lett. 572, 3–7. doi: 10.1016/j.febslet.2004.07.003 15304314

[B16] Breen S. Williams S. J. Outram M. Kobe B. Solomon P. S. (2017). Emerging insights into the functions of pathogenesis-related protein 1. Trends Plant Sci. 22, 871–879. doi: 10.1016/j.tplants.2017.06.013 28743380

[B17] Breen S. Williams S. J. Winterberg B. Kobe B. Solomon P. S. (2016). Wheat PR-1 proteins are targeted by necrotrophic pathogen effector proteins. Plant J. 88, 13–25. doi: 10.1111/tpj.13228 27258471

[B18] Cantacessi C. Gasser R. B. (2012). SCP/TAPS proteins in helminths–where to from now? Mol. Cell Probes 26, 54–59. doi: 10.1016/j.mcp.2011.10.001 22005034

[B19] Cesarino I. (2019). Structural features and regulation of lignin deposited upon biotic and abiotic stresses. Curr. Opin. Biotechnol. 56, 209–214. doi: 10.1016/j.copbio.2018.12.012 30684783

[B20] Chanclud E. Morel J. B. (2016). Plant hormones: a fungal point of view. Mol. Plant Pathol. 17, 1289–1297. doi: 10.1111/mpp.12393 26950404 PMC6638337

[B21] Chen Y. L. Lee C. Y. Cheng K. T. Chang W. H. Huang R. N. Nam H. G.. (2014). Quantitative peptidomics study reveals that a wound-induced peptide from PR-1 regulates immune signaling in tomato. Plant Cell 26, 4135–4148. doi: 10.1105/tpc.114.131185 25361956 PMC4247587

[B22] Chen Y. L. Lin F. W. Cheng K. T. Chang C. H. Hung S. C. Efferth T.. (2023). XCP1 cleaves pathogenesis-related protein 1 into CAPE9 for systemic immunity in Arabidopsis. Nat. Commun. 14, 4697. doi: 10.1038/s41467-023-40406-7 37542077 PMC10403534

[B23] Chen X. Zhang R. Takada A. Iwatani S. Oka C. Kitamoto T.. (2017). The role of Bgl2p in the transition to filamentous cells during biofilm formation by. Candida albicans. Mycoses 60, 96–103. doi: 10.1111/myc.12554 27597232

[B24] Choi D. S. Hwang I. S. Hwang B. K. (2012). Requirement of the cytosolic interaction between PATHOGENESIS-RELATED PROTEIN10 and LEUCINE-RICH REPEAT PROTEIN1 for cell death and defense signaling in pepper. Plant Cell 24, 1675–1690. doi: 10.1105/tpc.112.095869 22492811 PMC3398571

[B25] Christensen A. B. Cho B. H. Næsby M. Gregersen P. L. Brandt J. Madriz-Ordeñana K.. (2002). The molecular characterization of two barley proteins establishes the novel PR-17 family of pathogenesis-related proteins. Mol. Plant Pathol. 3, 135–144. doi: 10.1046/j.1364-3703.2002.00105.x 20569319

[B26] Ciuffetti L. M. Manning V. A. Pandelova I. Betts M. F. Martinez J. P. (2010). Host-selective toxins, Ptr ToxA and Ptr ToxB, as necrotrophic effectors in the Pyrenophora tritici-repentis-wheat interaction. New Phytol. 187, 911–919. doi: 10.1111/j.1469-8137.2010.03362.x 20646221

[B27] Coates M. E. Beynon J. L. (2010). Hyaloperonospora Arabidopsidis as a pathogen model. Annu. Rev. Phytopathol. 48, 329–345. doi: 10.1146/annurev-phyto-080508-094422 19400636

[B28] Darwiche R. Kelleher A. Hudspeth E. M. Schneiter R. Asojo O. A. (2016). Structural and functional characterization of the CAP domain of pathogen-related yeast 1 (Pry1) protein. Sci. Rep. 6, 28838. doi: 10.1038/srep28838 27344972 PMC4921858

[B29] Dean R. Van Kan J. A. Pretorius Z. A. Hammond-Kosack K. E. Di Pietro A. Spanu P. D.. (2012). The Top 10 fungal pathogens in molecular plant pathology. Mol. Plant Pathol. 13, 414–430. doi: 10.1111/j.1364-3703.2011.00783.x 22471698 PMC6638784

[B30] de Jesús-Pires C. Ferreira-Neto J. R. C. Pacifico Bezerra-Neto J. Kido E. A. de Oliveira Silva R. L. Pandolfi V.. (2020). Plant thaumatin-like proteins: Function, evolution and biotechnological applications. Curr. Protein Pept. Sci. 21, 36–51. doi: 10.2174/1389203720666190318164905 30887921

[B31] Dos Santos C. Franco O. L. (2023). Pathogenesis-related proteins (PRs) with enzyme activity activating plant defense responses. Plants (Basel) 12, 2226. doi: 10.3390/plants12112226 37299204 PMC10255391

[B32] Dunwell J. M. Purvis A. Khuri S. (2004). Cupins: the most functionally diverse protein superfamily. Phytochemistry 65, 7–17. doi: 10.1016/j.phytochem.2003.08.016 14697267

[B33] Edreva A. (2005). Pathogenesis-related proteins: research progress in the last 15 years. Gen. Appl. Plant Physiol. 31, 105–124.

[B34] El Hadrami A. Islam M. R. Adam L. R. Daayf F. (2015). A cupin domain-containing protein with a quercetinase activity (VdQase) regulates Verticillium dahliae’s pathogenicity and contributes to counteracting host defenses. Front. Plant Sci. 6. doi: 10.3389/fpls.2015.00440 PMC4462102 26113857

[B35] Epple P. Apel K. Bohlmann H. (1997). Overexpression of an endogenous thionin enhances resistance of Arabidopsis against. Fusarium oxysporum. Plant Cell 9, 509–520. doi: 10.1105/tpc.9.4.509 9144959 PMC156935

[B36] Fan H. Yang W. Nie J. Lin C. Wu J. Wu D.. (2021). Characterization of a secretory YML079-like cupin protein that contributes to sclerotinia sclerotiorum pathogenicity. Microorganisms 9, 2519. doi: 10.3390/microorganisms9122519 34946121 PMC8704077

[B37] Farvardin A. González-Hernández A. I. Llorens E. García-Agustín P. Scalschi L. Vicedo B. (2020). The apoplast: a key player in plant survival. Antioxidants (Basel) 9, 604. doi: 10.3390/antiox9070604 32664231 PMC7402137

[B38] Fernandes H. Michalska K. Sikorski M. Jaskolski M. (2013). Structural and functional aspects of PR-10 proteins. FEBS J. 280, 1169–1199. doi: 10.1111/febs.12114 23289796

[B39] Ferreira R. B. Monteiro S. Freitas R. Santos C. N. Chen Z. Batista L. M.. (2007). The role of plant defence proteins in fungal pathogenesis. Mol. Plant Pathol. 8, 677–700. doi: 10.1111/j.1364-3703.2007.00419.x 20507530

[B40] Figueiredo J. Sousa Silva M. Figueiredo A. (2018). Subtilisin-like proteases in plant defence: the past, the present and beyond. Mol. Plant Pathol. 19, 1017–1028. doi: 10.1111/mpp.12567 28524452 PMC6638164

[B41] Fones H. N. Bebber D. P. Chaloner T. M. Kay W. T. Steinberg G. Gurr S. J. (2020). Threats to global food security from emerging fungal and oomycete crop pathogens. Nat. Food 1, 332–342. doi: 10.1038/s43016-020-0075-0 37128085

[B42] Franco O. L. Rigden D. J. Melo F. R. Grossi-De-Sá M. F. (2002). Plant alpha-amylase inhibitors and their interaction with insect alpha-amylases. Eur. J. Biochem. 269, 397–412. doi: 10.1046/j.0014-2956.2001.02656.x 11856298

[B43] Fukamizo T. Shinya S. (2019). Chitin/chitosan-active enzymes involved in plant-microbe interactions. Adv. Exp. Med. Biol. 1142, 253–272. doi: 10.1007/978-981-13-7318-3_12 31102250

[B44] Gamir J. Darwiche R. Van’t Hof P. Choudhary V. Stumpe M. Schneiter R.. (2017). The sterol-binding activity of PATHOGENESIS-RELATED PROTEIN 1 reveals the mode of action of an antimicrobial protein. Plant J. 89, 502–509. doi: 10.1111/tpj.13398 27747953

[B45] Gao H. Ma K. Ji G. Pan L. Zhou Q. (2022). Lipid transfer proteins involved in plant-pathogen interactions and their molecular mechanisms. Mol. Plant Pathol. 23, 1815–1829. doi: 10.1111/mpp.13264 36052490 PMC9644281

[B46] Gianinazzi S. Martin C. Vallee J. C. (1970). Hypersensitivity to viruses, temperature and soluble proteins in *Nicotiana xanthi* n.c. appearance of new macromolecules at the repression of viral synthesis. C R Acad. Sci. Hebd Seances Acad. Sci. D 270, 2383–2386. 4997619

[B47] Gibbs G. M. Roelants K. O’Bryan M. K. (2008). The CAP superfamily: cysteine-rich secretory proteins, antigen 5, and pathogenesis-related 1 proteins–roles in reproduction, cancer, and immune defense. Endocr. Rev. 29, 865–897. doi: 10.1210/er.2008-0032 18824526

[B48] Gong B. Q. Wang F. Z. Li J. F. (2020). Hide-and-seek: Chitin-triggered plant immunity and fungal counterstrategies. Trends Plant Sci. 25, 805–816. doi: 10.1016/j.tplants.2020.03.006 32673581

[B49] Grenier J. Potvin C. Trudel J. Asselin A. (1999). Some thaumatin-like proteins hydrolyse polymeric beta-1,3-glucans. Plant J. 19, 473–480. doi: 10.1046/j.1365-313x.1999.00551.x 10504569

[B50] Griffith M. Yaish M. W. (2004). Antifreeze proteins in overwintering plants: a tale of two activities. Trends Plant Sci. 9, 399–405. doi: 10.1016/j.tplants.2004.06.007 15358271

[B51] Grosse-Holz F. M. van der Hoorn R. A. (2016). Juggling jobs: roles and mechanisms of multifunctional protease inhibitors in plants. New Phytol. 210, 794–807. doi: 10.1111/nph.13839 26800491

[B52] Grünwald N. J. Goss E. M. Press C. M. (2008). Phytophthora ramorum: a pathogen with a remarkably wide host range causing sudden oak death on oaks and ramorum blight on woody ornamentals. Mol. Plant Pathol. 9, 729–740. doi: 10.1111/j.1364-3703.2008.00500.x 19019002 PMC6640315

[B53] Guo J. Mou Y. Li Y. Yang Q. Wang X. Lin H.. (2023). Silencing a chitinase gene, PstChia1, reduces virulence of *Puccinia striiformis* f. sp. *tritici* . Int. J. Mol. Sci. 24, 8215. doi: 10.3390/ijms24098215 37175921 PMC10179651

[B54] Gust A. A. Felix G. (2014). Receptor like proteins associate with SOBIR1-type of adaptors to form bimolecular receptor kinases. Curr. Opin. Plant Biol. 21, 104–111. doi: 10.1016/j.pbi.2014.07.007 25064074

[B55] Hakim U. A. Hussain A. Shaban M. Khan A. H. Alariqi M. Gul S.. (2018). Osmotin: a plant defense tool against biotic and abiotic stresses. Plant Physiol. Biochem. 123, 149–159. doi: 10.1016/j.plaphy.2017.12.012 29245030

[B56] Han G. Z. (2019). Origin and evolution of the plant immune system. New Phytol. 222, 70–83. doi: 10.1111/nph.15596 30575972

[B57] Han Y. Song L. Peng C. Liu X. Liu L. Zhang Y.. (2019). A Magnaporthe chitinase interacts with a rice jacalin-related lectin to promote host colonization. Plant Physiol. 179, 1416–1430. doi: 10.1104/pp.18.01594 30696749 PMC6446787

[B58] Han Z. Xiong D. Schneiter R. Tian C. (2023). The function of plant PR1 and other members of the CAP protein superfamily in plant-pathogen interactions. Mol. Plant Pathol. 24, 651–668. doi: 10.1111/mpp.13320 36932700 PMC10189770

[B59] Han Z. Xiong D. Xu Z. Liu T. Tian C. (2021). The *Cytospora chrysosperma* virulence effector CcCAP1 mainly localizes to the plant nucleus to suppress plant immune responses. mSphere 6, e00883–e00820. doi: 10.1128/msphere.00883-20 33627507 PMC8544888

[B60] Henrissat B. Bairoch A. (1993). New families in the classification of glycosyl hydrolases based on amino acid sequence similarities. Biochem. J. 293, 781–788. doi: 10.1042/bj2930781 8352747 PMC1134435

[B61] Homma F. Huang J. van der Hoorn R. A. L. (2023). AlphaFold-Multimer predicts cross-kingdom interactions at the plant-pathogen interface. Nat. Commun. 14, 1–13. doi: 10.1038/s41467-023-41721-9 PMC10533508 37758696

[B62] Höng K. Austerlitz T. Bohlmann T. Bohlmann H. (2021). The thionin family of antimicrobial peptides. PloS One 16, e0254549. doi: 10.1371/journal.pone.0254549 34260649 PMC8279376

[B63] Islam M. M. El-Sappah A. H. Ali H. M. Zandi P. Huang Q. Soaud S. A.. (2023). Pathogenesis-related proteins (PRs) countering environmental stress in plants: a review. South Afr. J. Bot. 160, 414–427. doi: 10.1016/j.sajb.2023.07.003

[B64] Jain D. Khurana J. P. (2018). “Role of pathogenesis-Related (PR) proteins in plant microbes defence mechanism,” in Molecular aspects of plant-pathogen interactions. Eds. Singh A. Singh I. K. (Springer Nature, Singapore), 265–281.

[B65] Jiang H. Xia Y. Zhang S. Zhang Z. Feng H. Zhang Q.. (2023). The CAP superfamily protein PsCAP1 secreted by Phytophthora triggers immune responses in *Nicotiana benthamiana* through a leucine-rich repeat receptor-like protein. New Phytol. 240, 784–801. doi: 10.1111/nph.19194 37615219

[B66] Jones J. D. Dangl J. L. (2006). The plant immune system. Nature 444, 323–329. doi: 10.1038/nature05286 17108957

[B67] Joshi V. Joshi N. Vyas A. Jadhav S. K. (2021). Pathogenesis-related proteins: Role in plant defense. Biocontrol Agents Secondary Metabolites, 573–590. doi: 10.1016/B978-0-12-822919-4.00025-9

[B68] Kamoun S. Furzer O. Jones J. D. Judelson H. S. Ali G. S. Dalio R. J.. (2015). The Top 10 oomycete pathogens in molecular plant pathology. Mol. Plant Pathol. 16, 413–434. doi: 10.1111/mpp.12190 25178392 PMC6638381

[B69] Kirino H. Yoshimoto K. Shinya R. (2020). Thaumatin-like proteins and a cysteine protease inhibitor secreted by the pine wood nematode *Bursaphelenchus xylophilus* induce cell death in *Nicotiana benthamiana* . PloS One 15, e0241613. doi: 10.1371/journal.pone.0241613 33125444 PMC7598465

[B70] Klinter S. Bulone V. Arvestad L. (2019). Diversity and evolution of chitin synthases in oomycetes (Straminipila: Oomycota). Mol. Phylogenet Evol. 139, 106558. doi: 10.1016/j.ympev.2019.106558 31288106

[B71] Klosterman S. J. Atallah Z. K. Vallad G. E. Subbarao K. V. (2009). Diversity, pathogenicity, and management of verticillium species. Annu. Rev. Phytopathol. 47, 39–62. doi: 10.1146/annurev-phyto-080508-081748 19385730

[B72] Koiwa H. Kato H. Nakatsu T. Oda J. Yamada Y. Sato F. (1999). Crystal structure of tobacco PR-5d protein at 1.8 A resolution reveals a conserved acidic cleft structure in antifungal thaumatin-like proteins. J. Mol. Biol. 286, 1137–1145. doi: 10.1006/jmbi.1998.2540 10047487

[B73] Levy A. Guenoune-Gelbart D. Epel B. L. (2007). Beta-1,3-Glucanases: Plasmodesmal gate keepers for intercellular communication. Plant Signal Behav. 2, 404–407. doi: 10.4161/psb.2.5.4334 19704615 PMC2634228

[B74] Li P. Lu Y. J. Chen H. Day B. (2020). The lifecycle of the plant immune system. CRC Crit. Rev. Plant Sci. 39, 72–100. doi: 10.1080/07352689.2020.1757829 33343063 PMC7748258

[B75] Liang D. Andersen C. B. Vetukuri R. R. Dou D. Grenville-Briggs L. J. (2020). Horizontal Gene Transfer and Tandem Duplication Shape the Unique CAZyme Complement of the Mycoparasitic Oomycetes Pythium oligandrum and Pythium periplocum. Front. Microbiol. 11. doi: 10.3389/fmicb.2020.581698 PMC7720654 33329445

[B76] Liang X. Moomaw E. W. Rollins J. A. (2015). Fungal oxalate decarboxylase activity contributes to Sclerotinia sclerotiorum early infection by affecting both compound appressoria development and function. Mol. Plant Pathol. 16, 825–836. doi: 10.1111/mpp.12239 25597873 PMC6638544

[B77] Liebrand T. W. van den Burg H. A. Joosten M. H. (2014). Two for all: receptor-associated kinases SOBIR1 and BAK1. Trends Plant Sci. 19, 123–132. doi: 10.1016/j.tplants.2013.10.003 24238702

[B78] Lin Y. H. Xu M. Y. Hsu C. C. Damei F. A. Lee H. C. Tsai W. L.. (2023). Ustilago maydis PR-1-like protein has evolved two distinct domains for dual virulence activities. Nat. Commun. 14, 5755. doi: 10.1038/s41467-023-41459-4 37716995 PMC10505147

[B79] Liu Q. Luo L. Zheng L. (2018). Lignins: Biosynthesis and biological functions in plants. Int. J. Mol. Sci. 19, 335. doi: 10.3390/ijms19020335 29364145 PMC5855557

[B80] Liu X. Xie J. Fu Y. Jiang D. Chen T. Cheng J. (2020). The subtilisin-like protease bcser2 affects the sclerotial formation, conidiation and virulence of botrytis cinerea. Int. J. Mol. Sci. 21, 603. doi: 10.3390/ijms21020603 31963451 PMC7013506

[B81] Liu F. Zhang X. Lu C. Zeng X. Li Y. Fu D.. (2015). Non-specific lipid transfer proteins in plants: presenting new advances and an integrated functional analysis. J. Exp. Bot. 66, 5663–5681. doi: 10.1093/jxb/erv313 26139823

[B82] Longsaward R. Sanguankiattichai N. Viboonjun U. van der Hoorn R. A. L. (2023). Letter to the editor: cautionary note on ribonuclease activity of recombinant PR-10 proteins. Plant Cell Physiol. 64, 847–849. doi: 10.1093/pcp/pcad062 37319028 PMC10434734

[B83] Lozano-Torres J. L. Wilbers R. H. Gawronski P. Boshoven J. C. Finkers-Tomczak A. Cordewener J. H.. (2012). Dual disease resistance mediated by the immune receptor Cf-2 in tomato requires a common virulence target of a fungus and a nematode. Proc. Natl. Acad. Sci. U.S.A. 109, 10119–10124. doi: 10.1073/pnas.1202867109 22675118 PMC3382537

[B84] Lozano-Torres J. L. Wilbers R. H. Warmerdam S. Finkers-Tomczak A. Diaz-Granados A. van Schaik C. C.. (2014). Apoplastic venom allergen-like proteins of cyst nematodes modulate the activation of basal plant innate immunity by cell surface receptors. PloS Pathog. 10, e1004569. doi: 10.1371/journal.ppat.1004569 25500833 PMC4263768

[B85] Lu S. Edwards M. C. (2018). Molecular characterization and functional analysis of PR-1-like proteins identified from the wheat head blight fungus. Fusarium graminearum. Phytopathol. 108, 510–520. doi: 10.1094/PHYTO-08-17-0268-R 29117786

[B86] Lu S. Faris J. D. Sherwood R. Edwards M. C. (2013). Dimerization and protease resistance: new insight into the function of PR-1. J. Plant Physiol. 170, 105–110. doi: 10.1016/j.jplph.2012.08.006 22921679

[B87] Lu S. Faris J. D. Sherwood R. Friesen T. L. Edwards M. C. (2014). A dimeric PR-1-type pathogenesis-related protein interacts with ToxA and potentially mediates ToxA-induced necrosis in sensitive wheat. Mol. Plant Pathol. 15, 650–663. doi: 10.1111/mpp.12122 24433289 PMC6638811

[B88] McLaughlin J. E. Darwish N. I. Garcia-Sanchez J. Tyagi N. Trick H. N. McCormick S.. (2021). A lipid transfer protein has antifungal and antioxidant activity and suppresses fusarium head blight disease and DON accumulation in transgenic wheat. Phytopathology 111, 671–683. doi: 10.1094/PHYTO-04-20-0153-R 32896217

[B89] Mélida H. Sandoval-Sierra J. V. Diéguez-Uribeondo J. Bulone V. (2013). Analyses of extracellular carbohydrates in oomycetes unveil the existence of three different cell wall types. Eukaryot Cell 12, 194–203. doi: 10.1128/EC.00288-12 23204192 PMC3571302

[B90] Melnikova D. N. Finkina E. I. Bogdanov I. V. Tagaev A. A. Ovchinnikova T. V. (2022). Features and possible applications of plant lipid-binding and transfer proteins. Membranes (Basel) 13, 2. doi: 10.3390/membranes13010002 36676809 PMC9866449

[B91] Meng F. Li Y. Liu Z. Feng Y. Wang X. Zhang X. (2022). Expression of the thaumatin-like protein-1 gene (Bx-tlp-1) from pine wood nematode *Bursaphelenchus xylophilus* affects terpene metabolism in pine trees. Phytopathology 112, 888–897. doi: 10.1094/PHYTO-07-21-0289-R 35311527

[B92] Meng F. Li Y. Wang X. Feng Y. Liu Z. Zhang W.. (2019). Thaumatin-like protein-1 gene (Bx-tlp-1) is associated with the pathogenicity of *Bursaphelenchus xylophilus* . Phytopathology 109, 1949–1956. doi: 10.1094/PHYTO-03-19-0082-R 31573422

[B93] Menu-Bouaouiche L. Vriet C. Peumans W. J. Barre A. Van Damme E. J. Rougé P. (2003). A molecular basis for the endo-beta 1,3-glucanase activity of the thaumatin-like proteins from edible fruits. Biochimie 85, 123–131. doi: 10.1016/s0300-9084(03)00058-0 12765782

[B94] Mir A. A. Park S. Y. Abu Sadat M. Kim S. Choi J. Jeon J.. (2015). Systematic characterization of the peroxidase gene family provides new insights into fungal pathogenicity in. Magnaporthe oryzae. Sci. Rep. 5, 11831. doi: 10.1038/srep11831 26134974 PMC4488832

[B95] Missaoui K. Gonzalez-Klein Z. Pazos-Castro D. Hernandez-Ramirez G. Garrido-Arandia M. Brini F.. (2022). Plant non-specific lipid transfer proteins: an overview. Plant Physiol. Biochem. 171, 115–127. doi: 10.1016/j.plaphy.2021.12.026 34992048

[B96] Mitsuhara I. Iwai T. Seo S. Yanagawa Y. Kawahigasi H. Hirose S.. (2008). Characteristic expression of twelve rice PR1 family genes in response to pathogen infection, wounding, and defense-related signal compounds (121/180). Mol. Genet. Genomics 279, 415–427. doi: 10.1007/s00438-008-0322-9 18247056 PMC2270915

[B97] Mittler R. (2002). Oxidative stress, antioxidants and stress tolerance. Trends Plant Sci. 7, 405–410. doi: 10.1016/s1360-1385(02)02312-9 12234732

[B98] Monod M. Capoccia S. Léchenne B. Zaugg C. Holdom M. Jousson O. (2002). Secreted proteases from pathogenic fungi. Int. J. Med. Microbiol. 292, 405–419. doi: 10.1078/1438-4221-00223 12452286

[B99] Monzingo A. F. Marcotte E. M. Hart P. J. Robertus J. D. (1996). Chitinases, chitosanases, and lysozymes can be divided into procaryotic and eucaryotic families sharing a conserved core. Nat. Struct. Biol. 3, 133–140. doi: 10.1038/nsb0296-133 8564539

[B100] Ngou B. P. M. Ding P. Jones J. D. G. (2022). Thirty years of resistance: Zig-zag through the plant immune system. Plant Cell 34, 1447–1478. doi: 10.1093/plcell/koac041 35167697 PMC9048904

[B101] Niderman T. Genetet I. Bruyere T. Gees R. Stintzi A. Legrand M.. (1995). Pathogenesis-related PR-1 proteins are antifungal. Isolation and characterization of three 14-kilodalton proteins of tomato and of a basic PR-1 of tobacco with inhibitory activity against. Phytophthora infestans. Plant Physiol. 108, 17–27. doi: 10.1104/pp.108.1.17 7784503 PMC157301

[B102] Ohnuma T. Umemoto N. Nagata T. Shinya S. Numata T. Taira T.. (2014). Crystal structure of a “loopless” GH19 chitinase in complex with chitin tetrasaccharide spanning the catalytic center. Biochim. Biophys. Acta 1844, 793–802. doi: 10.1016/j.bbapap.2014.02.013 24582745

[B103] Okushima Y. Koizumi N. Kusano T. Sano H. (2000). Secreted proteins of tobacco cultured BY2 cells: identification of a new member of pathogenesis-related proteins. Plant Mol. Biol. 42, 479–488. doi: 10.1023/a:1006393326985 10798617

[B104] Oyeleye A. Normi Y. M. (2018). Chitinase: diversity, limitations, and trends in engineering for suitable applications. Biosci. Rep. 38, BSR2018032300. doi: 10.1042/BSR20180323 30042170 PMC6131217

[B105] Parisi K. Shafee T. M. A. Quimbar P. van der Weerden N. L. Bleackley M. R. Anderson M. A. (2019). The evolution, function and mechanisms of action for plant defensins. Semin. Cell Dev. Biol. 88, 107–118. doi: 10.1016/j.semcdb.2018.02.004 29432955

[B106] Passardi F. Penel C. Dunand C. (2004). Performing the paradoxical: how plant peroxidases modify the cell wall. Trends Plant Sci. 9, 534–540. doi: 10.1016/j.tplants.2004.09.002 15501178

[B107] Pedro H. Maheswari U. Urban M. Irvine A. G. Cuzick A. McDowall M. D.. (2016). PhytoPath: an integrative resource for plant pathogen genomics. Nucleic Acids Res. 44, D688–D693. doi: 10.1093/nar/gkv1052 26476449 PMC4702788

[B108] Perrot T. Pauly M. Ramírez V. (2022). Emerging roles of β-glucanases in plant development and adaptative responses. Plants (Basel) 11, 1119. doi: 10.3390/plants11091119 35567119 PMC9099982

[B109] Pettersen E. F. Goddard T. D. Huang C. C. Couch G. S. Greenblatt D. M. Meng E. C.. (2004). UCSF Chimera–a visualization system for exploratory research and analysis. J. Comput. Chem. 25, 1605–1612. doi: 10.1002/jcc.20084 15264254

[B110] Poria V. Rana A. Kumari A. Grewal J. Pranaw K. Singh S. (2021). Current perspectives on chitinolytic enzymes and their agro-industrial applications. Biol. (Basel) 10, 1319. doi: 10.3390/biology10121319 PMC8698876 34943233

[B111] Prados-Rosales R. C. Roldan-Rodriguez R. Serena C. Lopez-Berges M. S. Guarro J. Martinez-del-Pozo A.. (2012). A PR-1-like protein of *Fusarium oxysporum* functions in virulence on mammalian hosts. J. Biol. Chem. 287, 21970–21979. doi: 10.1074/jbc.M112.364034 22553200 PMC3381157

[B112] Radauer C. Lackner P. Breiteneder H. (2008). The Bet v 1 fold: an ancient, versatile scaffold for binding of large, hydrophobic ligands. BMC Evol. Biol. 8, 286. doi: 10.1186/1471-2148-8-286 18922149 PMC2577659

[B113] Rawlings N. D. Barrett A. J. Thomas P. D. Huang X. Bateman A. Finn R. D. (2018). The MEROPS database of proteolytic enzymes, their substrates and inhibitors in 2017 and a comparison with peptidases in the PANTHER database. Nucleic Acids Res. 46, D624–D632. doi: 10.1093/nar/gkx1134 29145643 PMC5753285

[B114] Riviere M. P. Marais A. Ponchet M. Willats W. Galiana E. (2008). Silencing of acidic pathogenesis-related PR-1 genes increases extracellular beta-(1-3)-glucanase activity at the onset of tobacco defence reactions. J. Exp. Bot. 59, 1225–1239. doi: 10.1093/jxb/ern044 18390849

[B115] Roberts W. K. Selitrennikoff C. P. (1990). Zeamatin, an antifungal protein from maize with membrane-permeabilizing activity. J. Gen. Microbiolol. 136, 1771–1778. doi: 10.1099/00221287-136-9-1771

[B116] Ryan C. A. (1989). Proteinase inhibitor gene families: strategies for transformation to improve plant defenses against herbivores. Bioessays 10, 20–24. doi: 10.1002/bies.950100106 2653308

[B117] Sakamoto Y. Watanabe H. Nagai M. Nakade K. Takahashi M. Sato T. (2006). Lentinula edodes tlg1 encodes a thaumatin-like protein that is involved in lentinan degradation and fruiting body senescence. Plant Physiol. 141, 793–801. doi: 10.1104/pp.106.076679 16648221 PMC1475445

[B118] Sarthy A. V. McGonigal T. Coen M. Frost D. J. Meulbroek J. A. Goldman R. C. (1997). Phenotype in *Candida albicans* of a disruption of the BGL2 gene encoding a 1,3-beta-glucosyltransferase. Microbiol. (Reading) 143, 367–376. doi: 10.1099/00221287-143-2-367 9043114

[B119] Schaller A. Stintzi A. Rivas S. Serrano I. Chichkova N. V. Vartapetian A. B.. (2018). From structure to function–a family portrait of plant subtilases. New Phytol. 218, 901–915. doi: 10.1111/nph.14582 28467631

[B120] Sels J. Mathys J. De Coninck B. M. Cammue B. P. De Bolle M. F. (2008). Plant pathogenesis-related (PR) proteins: a focus on PR peptides. Plant Physiol. Biochem. 46, 941–950. doi: 10.1016/j.plaphy.2008.06.011 18674922

[B121] Sharma A. Sharma H. Rajput R. Pandey A. Upadhyay S. K. (2021). Molecular characterization revealed the role of thaumatin-like proteins of bread wheat in stress response. Front. Plant Sci. 12. doi: 10.3389/fpls.2021.807448 PMC8786798 35087559

[B122] Shi L. Li R. Liao S. Bai L. Lu Q. Chen B. (2014). Prb1, a subtilisin-like protease, is required for virulence and phenotypical traits in the chestnut blight fungus. FEMS Microbiol. Lett. 359, 26–33. doi: 10.1111/1574-6968.12547 25066598

[B123] Singh B. K. Delgado-Baquerizo M. Egidi E. Guirado E. Leach J. E. Liu H.. (2023). Climate change impacts on plant pathogens, food security and paths forward. Nat. Rev. Microbiol. 21, 640–656. doi: 10.1038/s41579-023-00900-7 PMC10153038 37131070

[B124] Srinivas C. Nirmala Devi D. Narasimha Murthy K. Mohan C. D. Lakshmeesha T. R. Singh B.. (2019). *Fusarium oxysporum* f. sp. *lycopersici* causal agent of vascular wilt disease of tomato: Biology to diversity- A review. Saudi J. Biol. Sci. 26, 1315–1324. doi: 10.1016/j.sjbs.2019.06.002 31762590 PMC6864208

[B125] Stec B. (2006). Plant thionins–the structural perspective. Cell Mol. Life Sci. 63, 1370–1385. doi: 10.1007/s00018-005-5574-5 16715411 PMC11136031

[B126] Stintzi A. Heitz T. Prasad V. Wiedemann-Merdinoglu S. Kauffmann S. Geoffroy P.. (1993). Plant ‘pathogenesis-related’ proteins and their role in defense against pathogens. Biochimie 75, 687–706. doi: 10.1016/0300-9084(93)90100-7 8286442

[B127] Sung Y. C. Outram M. A. Breen S. Wang C. Dagvadorj B. Winterberg B.. (2021). PR1-mediated defence via C-terminal peptide release is targeted by a fungal pathogen effector. New Phytol. 229, 3467–3480. doi: 10.1111/nph.17128 33277705

[B128] Tam J. P. Wang S. Wong K. H. Tan W. L. (2015). Antimicrobial peptides from plants. Pharm. (Basel) 8, 711–757. doi: 10.3390/ph8040711 PMC4695807 26580629

[B129] Tan J. Zhao H. Li J. Gong Y. Li X. (2023). The devastating rice blast airborne pathogen magnaporthe oryzae-A review on genes studied with mutant analysis. Pathogens 12, 379. doi: 10.3390/pathogens12030379 36986301 PMC10055536

[B130] Tanaka K. Heil M. (2021). Damage-associated molecular patterns (DAMPs) in plant innate immunity: applying the danger model and evolutionary perspectives. Annu. Rev. Phytopathol. 59, 53–75. doi: 10.1146/annurev-phyto-082718-100146 33900789

[B131] Teixeira P. J. P. Colaianni N. R. Fitzpatrick C. R. Dangl J. L. (2019). Beyond pathogens: microbiota interactions with the plant immune system. Curr. Opin. Microbiol. 49, 7–17. doi: 10.1016/j.mib.2019.08.003 31563068

[B132] Teixeira P. J. Thomazella D. P. Vidal R. O. do Prado P. F. Reis O. Baroni R. M.. (2012). The fungal pathogen *Moniliophthora perniciosa* has genes similar to plant PR-1 that are highly expressed during its interaction with cacao. PloS One 7, e45929. doi: 10.1371/journal.pone.0045929 23029323 PMC3447762

[B133] Terras F. R. Eggermont K. Kovaleva V. Raikhel N. V. Osborn R. W. Kester A.. (1995). Small cysteine-rich antifungal proteins from radish: their role in host defense. Plant Cell 7, 573–588. doi: 10.1105/tpc.7.5.573 7780308 PMC160805

[B134] Terras F. Schoofs H. Thevissen K. Osborn R. W. Vanderleyden J. Cammue B.. (1993). Synergistic enhancement of the antifungal activity of wheat and barley thionins by radish and oilseed rape 2S albumins and by barley trypsin inhibitors. Plant Physiol. 103, 1311–1319. doi: 10.1104/pp.103.4.1311 12232024 PMC159121

[B135] Thevissen K. Cammue B. P. Lemaire K. Winderickx J. Dickson R. C. Lester R. L.. (2000). A gene encoding a sphingolipid biosynthesis enzyme determines the sensitivity of *Saccharomyces cerevisiae* to an antifungal plant defensin from dahlia (*Dahlia merckii*). Proc. Natl. Acad. Sci. U.S.A. 97, 9531–9536. doi: 10.1073/pnas.160077797 10931938 PMC16899

[B136] Trudel J. Grenier J. Potvin C. Asselin A. (1998). Several thaumatin-like proteins bind to beta-1,3-glucans. Plant Physiol. 118, 1431–1438. doi: 10.1104/pp.118.4.1431 9847118 PMC34760

[B137] van Loon L. C. Gerritsen Y. A. M. Ritter C. E. (1987). Identification, purification, and characterization of pathogenesis-related proteins from virus-infected Samsun NN tobacco leaves. Plant Mol. Biol. 9, 593–609. doi: 10.1007/BF00020536 24277196

[B138] van Loon L. C. Rep M. Pieterse C. M. (2006). Significance of inducible defense-related proteins in infected plants. Annu. Rev. Phytopathol. 44, 135–162. doi: 10.1146/annurev.phyto.44.070505.143425 16602946

[B139] van Loon L. C. van Kammen A. (1970). Polyacrylamide disc electrophoresis of the soluble leaf proteins from *Nicotiana tabacum* var. “Samsun” and “Samsun NN”. II. Changes in protein constitution after infection with tobacco mosaic virus. Virology 40, 190–211. doi: 10.1016/0042-6822(70)90395-8 4191688

[B140] Van Loon L. C. Van Strien E. A. (1999). The families of pathogenesis-related proteins, their activities, and comparative analysis of PR-1 type proteins. Physiol. Mol. Plant Pathol. 55, 85–97. doi: 10.1006/pmpp.1999.0213

[B141] Vlot A. C. Dempsey D. A. Klessig D. F. (2009). Salicylic acid, a multifaceted hormone to combat disease. Annu. Rev. Phytopathol. 47, 177–206. doi: 10.1146/annurev.phyto.050908.135202 19400653

[B142] Wang F. Shen S. Zhao C. Cui Z. Meng L. Wu W.. (2022). TaPR1 interacts with TaTLP1 via the αIV helix to be involved in wheat defense to *Puccinia triticina* through the CAPE1 motif. Front. Plant Sci. 13. doi: 10.3389/fpls.2022.874654 PMC9199852 35720612

[B143] Wang C. Yin Z. Nie J. Lin Y. Huang L. (2021). Identification and virulence analysis of CAP superfamily genes in *Valsa Mali* . Scientia Agricultura Sin. 54, 3440–3450. doi: 10.3864/j.issn.0578-1752.2021.16.007

[B144] Wang F. Yuan S. Wu W. Yang Y. Cui Z. Wang H.. (2020). TaTLP1 interacts with TaPR1 to contribute to wheat defense responses to leaf rust fungus. PloS Genet. 16, e1008713. doi: 10.1371/journal.pgen.1008713 32658889 PMC7357741

[B145] Wanke A. Malisic M. Wawra S. Zuccaro A. (2021). Unraveling the sugar code: the role of microbial extracellular glycans in plant-microbe interactions. J. Exp. Bot. 72, 15–35. doi: 10.1093/jxb/eraa414 32929496 PMC7816849

[B146] Westrick N. M. Smith D. L. Kabbage M. (2021). Disarming the host: detoxification of plant defense compounds during fungal necrotrophy. Front. Plant Sci. 12. doi: 10.3389/fpls.2021.651716 PMC8120277 33995447

[B147] Whisson S. C. Boevink P. C. Wang S. Birch P. R. (2016). The cell biology of late blight disease. Curr. Opin. Microbiol. 34, 127–135. doi: 10.1016/j.mib.2016.09.002 27723513 PMC5340842

[B148] Wilbers R. H. P. Schneiter R. Holterman M. H. M. Drurey C. Smant G. Asojo O. A.. (2018). Secreted venom allergen-like proteins of helminths: Conserved modulators of host responses in animals and plants. PloS Pathog. 14, e1007300. doi: 10.1371/journal.ppat.1007300 30335852 PMC6193718

[B149] Xu L. Wang H. Zhang C. Wang J. Chen A. Chen Y.. (2020). System-wide characterization of subtilases reveals that subtilisin-like protease FgPrb1 of Fusarium graminearum regulates fungal development and virulence. Fungal Genet. Biol. 144, 103449. doi: 10.1016/j.fgb.2020.103449 32890707

[B150] Yan T. Zhou X. Li J. Li G. Zhao Y. Wang H.. (2022). FoCupin1, a Cupin_1 domain-containing protein, is necessary for the virulence of Fusarium oxysporum f. sp. cubense tropical race 4. Front. Microbiol. 13. doi: 10.3389/fmicb.2022.1001540 PMC9468701 36110302

[B151] Yang H. Kim X. Skłenar J. Aubourg S. Sancho-Andrés G. Stahl E.. (2023). Subtilase-mediated biogenesis of the expanded family of SERINE RICH ENDOGENOUS PEPTIDES. Nat. Plants 9, 2085–2094. doi: 10.1038/s41477-023-01583-x 38049516

[B152] Yu C. Qi J. Han H. Wang P. Liu C. (2023). Progress in pathogenesis research of Ustilago maydis, and the metabolites involved along with their biosynthesis. Mol. Plant Pathol. 24, 495–509. doi: 10.1111/mpp.13307 36808861 PMC10098057

[B153] Zhang H. Mukherjee M. Kim J. E. Yu W. Shim W. B. (2018a). Fsr1, a striatin homologue, forms an endomembrane-associated complex that regulates virulence in the maize pathogen. Fusarium verticillioides. Mol. Plant Pathol. 19, 812–826. doi: 10.1111/mpp.12562 28467007 PMC6638083

[B154] Zhang J. Wang F. Liang F. Zhang Y. Ma L. Wang H.. (2018b). Functional analysis of a pathogenesis-related thaumatin-like protein gene TaLr35PR5 from wheat induced by leaf rust fungus. BMC Plant Biol. 18, 76. doi: 10.1186/s12870-018-1297-2 29728059 PMC5935958

[B155] Zhao J. Bi W. Zhao S. Su J. Li M. Ma L.. (2021). Wheat apoplast-localized lipid transfer protein TaLTP3 enhances defense responses against. Puccinia triticina. Front. Plant Sci. 12. doi: 10.3389/fpls.2021.771806 PMC8657149 34899796

[B156] Zhao M. Zhang Y. Guo H. Gan P. Cai M. Kang Z.. (2023). Identification and functional analysis of CAP genes from the wheat stripe rust fungus *Puccinia striiformis* f. sp. *tritici* . J. Fungi (Basel) 9, 734. doi: 10.3390/jof9070734 37504723 PMC10381272

[B157] Zhou J. M. Zhang Y. (2020). Plant immunity: Danger perception and signaling. Cell 181, 978–989. doi: 10.1016/j.cell.2020.04.028 32442407

[B158] Zribi I. Ghorbel M. Brini F. (2021). Pathogenesis related proteins (PRs): From cellular mechanisms to plant defense. Curr. Protein Pept. Sci. 22, 396–412. doi: 10.2174/1389203721999201231212736 33390143

