# Supplementary material for: Dual functionality of pathogenesis-related proteins: defensive role in plants versus immunosuppressive role in pathogens
Source: Front Plant Sci. 2024 Aug 2;15:1368467. doi: 10.3389/fpls.2024.1368467 (PMC11327054; doi:10.3389/fpls.2024.1368467)
Supplement: Supplementary file 1 [file DataSheet_1.pdf]

Supplementary Tables

Table S1. Identifiers of PR-like Genes from Filamentous Phytopathogens

Family	Pfam	Activity	*Magnaporthe oryzae*	*Botrytis cinerea*	*Puccinia striiformis*	*Fusarium oxysporum*	*Verticillium dahliae*	*Ustilago maydis*	*Phytophthora infestans*	*Hyaloperonospora arabidopsidis*	*Phytophthora ramorum*
**PR1**	PF00188	Immune signaling, Lipid-binding	MGG_03085<br>MGG_07807<br>MGG_06772<br>MGG_13936<br>MGG_03755<br>MGG_05100	BCIN_08g02680<br>BCIN_11g04730<br>BCIN_01g09180<br>BCIN_14g00420	PSTG_01738<br>PSTG_01739<br>PSTG_13342<br>PSTG_11017<br>PSTG_15884<br>PSTG_06516	FOXG_00482<br>FOXG_09795<br>FOXG_10300<br>FOXG_12292<br>FOXG_12428<br>FOXG_14109<br>FOXG_06245	VDAG_09343<br>VDAG_02733<br>VDAG_03487<br>VDAG_04828	UMAG_01204<br>UMAG_04343	Phyinf117174<br>Phyinf14883<br>Phyinf14926<br>Phyinf14946<br>Phyinf14947<br>Phyinf14949<br>Phyinf14951<br>Phyinf14952<br>Phyinf14953<br>Phyinf14954<br>Phyinf14955<br>Phyinf14967<br>Phyinf14970<br>Phyinf16418<br>Phyinf16419<br>Phyinf16420<br>Phyinf16421<br>Phyinf18884<br>Phyinf19703<br>Phyinf111612<br>Phyinf111613<br>Phyinf111616<br>Phyinf112481<br>Phyinf112482<br>Phyinf112483<br>Phyinf14566<br>Phyinf14567<br>Phyinf14568<br>Phyinf14569	Hyaar151<br>Hyaar153<br>Hyaar18924<br>Hyaar1858<br>Hyaar1859<br>Hyaar110787<br>Hyaar110788<br>Hyaar110789<br>Hyaar111490<br>Hyaar112558<br>Hyaar112559<br>Hyaar1802<br>Hyaar1803	Phyra1_182067<br>Phyra1_182077<br>Phyra1_182078<br>Phyra1_182080<br>Phyra1_182082<br>Phyra1_173685<br>Phyra1_173684<br>Phyra1_173686<br>Phyra1_174540<br>Phyra1_178264<br>Phyra1_178267<br>Phyra1_178919<br>Phyra1_196220<br>Phyra1_196231<br>Phyra1_180241<br>Phyra1_180240<br>Phyra1_180242<br>Phyra1_180475<br>Phyra1_195732<br>Phyra1_196230<br>Phyra1_174818
**PR2**	PF00332	β-1,3-glucanase	MGG_04689<br>MGG_06023<br>MGG_10400	BCIN_03g03840<br>BCIN_08g00910<br>BCIN_01g11220<br>BCIN_02g06910	PSTG_16373<br>PSTG_16375	FOXG_15659<br>FOXG_03723<br>FOXG_16943<br>FOXG_06041	VDAG_00511<br>VDAG_07185<br>VDAG_09510<br>VDAG_01689<br>VDAG_02343<br>VDAG_02469	UMAG_06078<br>UMAG_06133	Phyinf1203<br>Phyinf113636<br>Phyinf113643<br>Phyinf113647<br>Phyinf113651<br>Phyinf113664<br>Phyinf114264	Hyaar18547<br>Hyaar1781<br>Hyaar114284	Phyra1_185261<br>Phyra1_172319<br>Phyra1_172578<br>Phyra1_174267<br>Phyra1_180072<br>Phyra1_181533<br>Phyra1_181535
**PR3**	PF00182	Chitinase	/	/	/	/	/	/	Phyinf112400<br>Phyinf115211<br>Phyinf115213	Hyaar13729	Phyra1_171328
**PR4**	PF00967	Chitinase	/	/	/	/	/	/	/	/	/
**PR5**	PF00314	Thaumatin	MGG_03044	BCIN_16g04140<br>BCIN_09g04150	PSTG_11165<br>PSTG_11429<br>PSTG_11430<br>PSTG_16715	FOXG_03360	VDAG_01910	UMAG_03807	Phyinf1803<br>Phyinf18660	/	/
**PR6**	PF00280	Protease inhibitor	/	/	/	/	/	/	/	/	/
**PR7**	PF00082	Subtilisin-like endoprotease	MGG_10449<br>MGG_08966<br>MGG_07965<br>MGG_03670<br>MGG_02863<br>MGG_15291<br>MGG_09246<br>MGG_04733<br>MGG_07358<br>MGG_16831<br>MGG_14860<br>MGG_09990<br>MGG_02649<br>MGG_10445<br>MGG_08415<br>MGG_08429<br>MGG_08436<br>MGG_03316<br>MGG_00282<br>MGG_03870<br>MGG_09352<br>MGG_09817<br>MGG_02531<br>MGG_09073<br>MGG_07404<br>MGG_07559<br>MGG_13977<br>MGG_00940<br>MGG_09322<br>MGG_03056	BCIN_10g02530<br>BCIN_08g02990<br>BCIN_05g03290<br>BCIN_06g01370<br>BCIN_16g04460<br>BCIN_05g05830<br>BCIN_15g03150<br>BCIN_03g02040<br>BCIN_08g01020<br>BCIN_06g01010<br>BCIN_06g00620<br>BCIN_06g00330<br>BCIN_15g04670	PSTG_10928<br>PSTG_14252<br>PSTG_04600<br>PSTG_05113<br>PSTG_00912<br>PSTG_00927<br>PSTG_08707<br>PSTG_09271<br>PSTG_14941<br>PSTG_04216<br>PSTG_04259<br>PSTG_04264<br>PSTG_00696<br>PSTG_00915<br>PSTG_00930<br>PSTG_00932	FOXG_01145<br>FOXG_01284<br>FOXG_08795<br>FOXG_09801<br>FOXG_12263<br>FOXG_13463<br>FOXG_03262<br>FOXG_17011<br>FOXG_04749<br>FOXG_04896<br>FOXG_05775<br>FOXG_09594<br>FOXG_12879<br>FOXG_13295<br>FOXG_18343<br>FOXG_14511<br>FOXG_14793<br>FOXG_02695<br>FOXG_22424<br>FOXG_16982<br>FOXG_04700<br>FOXG_05860<br>FOXG_06062<br>FOXG_19284<br>FOXG_19376<br>FOXG_19497<br>FOXG_19498<br>FOXG_08084<br>FOXG_19740<br>FOXG_02380<br>FOXG_14564<br>FOXG_13604	VDAG_06121<br>VDAG_05709<br>VDAG_05967<br>VDAG_07176<br>VDAG_02670<br>VDAG_01043<br>VDAG_05299<br>VDAG_06012<br>VDAG_07131<br>VDAG_02277<br>VDAG_10474<br>VDAG_07367<br>VDAG_07373<br>VDAG_08100<br>VDAG_09626<br>VDAG_03905<br>VDAG_04865<br>VDAG_03685	UMAG_04400<br>UMAG_02843<br>UMAG_12106<br>UMAG_03024<br>UMAG_06118	Phyinf116981<br>Phyinf1625<br>Phyinf11200<br>Phyinf117677<br>Phyinf117751<br>Phyinf17249<br>Phyinf17250<br>Phyinf19522<br>Phyinf113152<br>Phyinf113259<br>Phyinf115494<br>Phyinf113880<br>Phyinf18629	Hyaar11391<br>Hyaar12216<br>Hyaar15496<br>Hyaar15686<br>Hyaar16367<br>Hyaar18309	Phyra1_184081<br>Phyra1_184204<br>Phyra1_138840<br>Phyra1_173616<br>Phyra1_179030<br>Phyra1_179126<br>Phyra1_195546

**PR8/11**	PF00704	Chitinase	MGG_10333<br>MGG_04534<br>MGG_01336<br>MGG_03599<br>MGG_05533<br>MGG_04732<br>MGG_01247<br>MGG_11231<br>MGG_01876<br>MGG_08054<br>MGG_07927<br>MGG_08458<br>MGG_17153<br>MGG_00086<br>MGG_17552<br>MGG_04073	BCIN_16g01590<br>BCIN_07g00890<br>BCIN_03g03220<br>BCIN_05g02660<br>BCIN_07g00160<br>BCIN_02g00570<br>BCIN_08g07050	PSTG_13182<br>PSTG_14802<br>PSTG_03810<br>PSTG_03812<br>PSTG_03813<br>PSTG_03991<br>PSTG_04016<br>PSTG_15142<br>PSTG_15161<br>PSTG_16340<br>PSTG_16360<br>PSTG_00935<br>PSTG_07122<br>PSTG_07135<br>PSTG_08478	FOXG_10827<br>FOXG_14329<br>FOXG_15329<br>FOXG_15151<br>FOXG_22639<br>FOXG_22648<br>FOXG_00277<br>FOXG_00921<br>FOXG_09583<br>FOXG_10034<br>FOXG_10748<br>FOXG_11492<br>FOXG_12653<br>FOXG_12882<br>FOXG_12492<br>FOXG_14047<br>FOXG_14840<br>FOXG_15473<br>FOXG_03402<br>FOXG_16128<br>FOXG_16251<br>FOXG_16430<br>FOXG_17257<br>FOXG_17332<br>FOXG_06883<br>FOXG_08721<br>FOXG_15373<br>FOXG_17685	VDAG_00322<br>VDAG_05658<br>VDAG_06206<br>VDAG_06734<br>VDAG_06825<br>VDAG_00901<br>VDAG_08741<br>VDAG_09205<br>VDAG_09560<br>VDAG_02162<br>VDAG_02356<br>VDAG_02396<br>VDAG_10493<br>VDAG_04416<br>VDAG_04782<br>VDAG_04833<br>VDAG_04879<br>VDAG_07000	UMAG_02758<br>UMAG_10419<br>UMAG_06190	Phyinf117427<br>Phyinf113414	Hyaar19540<br>Hyaar114252	Phyra1_181952<br>Phyra1_177376
**PR9**	PF00141	Heme-containing peroxidase	MGG_08200<br>MGG_04545<br>MGG_07790<br>MGG_00461<br>MGG_10877<br>MGG_10368<br>MGG_04337<br>MGG_14940<br>MGG_09398<br>MGG_09834	BCIN_03g07850<br>BCIN_07g05810<br>BCIN_13g03680<br>BCIN_01g09360	PSTG_07052<br>PSTG_07189	FOXG_00142<br>FOXG_10495<br>FOXG_12260<br>FOXG_13439<br>FOXG_13788<br>FOXG_14234<br>FOXG_17106<br>FOXG_17130<br>FOXG_17180<br>FOXG_17460	VDAG_01449<br>VDAG_00782<br>VDAG_06204<br>VDAG_10405<br>VDAG_02834<br>VDAG_03116<br>VDAG_04826	UMAG_01947<br>UMAG_02377<br>UMAG_11067	Phyinf1450<br>Phyinf11442<br>Phyinf116039<br>Phyinf116040	Hyaar110151<br>Hyaar14038<br>Hyaar18166<br>Hyaar18190	Phyra1_151170<br>Phyra1_186067<br>Phyra1_138638<br>Phyra1_173141<br>Phyra1_173140<br>Phyra1_177036
**PR10**	PF00407	Ribonuclease-like	/	/	/	/	/	/	/	/	/
**PR12**	PF00304	Plant defensin	/	/	/	/	/	/	/	/	/
**PR13**	PF00321	Thionin	/	/	/	/	/	/	/	/	/
**PR14**	PF00234	Non-specific lipid-transfer protein	/	/	/	/	/	/	/	/	/
**PR15/16**	PF00190	Oxalate oxidase	MGG_07824	BCIN_16g03240<br>BCIN_01g03150<br>BCIN_04g05650	/	FOXG_08602<br>FOXG_10358<br>FOXG_11962<br>FOXG_13375<br>FOXG_08770<br>FOXG_09843<br>FOXG_13201	VDAG_00088<br>VDAG_07656<br>VDAG_09056<br>VDAG_09019<br>VDAG_09251<br>VDAG_04026	/	/	/	/
**PR17**	PF04450	Putative aminopeptidase	MGG_04184	BCIN_07g01400	/	FOXG_08519	VDAG_00452	/	/	/	/

The table gives gene identifiers of PR-like proteins that are present in fungal (left-hand part) and oomycetes (right-hand part) phytopathogens. /, indicates that there is no gene present that matches the annotation of the respective plant PR protein family. PR-like families that are absent from the genomes of fungal or oomycetes are shaded in light green, i.e., PR3-, PR4-, PR6-, PR10-, PR12-, PR13-, PR14-, PR17-like. Genes were identified by screening sequences of individual plant PR family members against the genome sequences of *Magnaporthe oryzae* (*Pyricularia oryzae* 70-15, v3.0), *B. cinerea* (B05.10), *Puccinia* spp. (*Puccinia striiformis* f.sp. *tritici* PST-78, v1.0), *Fusarium oxysporum* (*Fusarium oxysporum* f.sp. *lycopersici* 4287, v2), *Verticillium dahliae* (VdLs.17), *Ustilago maydis* (521, v2.0), *Phytophthora infestans* (T30-4), *Hyaloperonospora arabidopsidis* (Emoy2, v2.0), and *Phytophthora ramorum* (v1.1) in the PhytoPath database (https://phytopathdb.org/) (Pedro et al., 2016).
